# Tetramethylpyrazine promotes stroke recovery by inducing the restoration of neurovascular unit and transformation of A1/A2 reactive astrocytes

**DOI:** 10.3389/fncel.2023.1125412

**Published:** 2023-03-27

**Authors:** Xue-feng Feng, Ming-cong Li, Zi-yue Lin, Man-zhong Li, Yun Lu, Yu-ming Zhuang, Jian-feng Lei, Lei Wang, Hui Zhao

**Affiliations:** ^1^School of Traditional Chinese Medicine, Capital Medical University, Beijing, China; ^2^Beijing Key Lab of TCM Collateral Disease Theory Research, Beijing, China; ^3^Medical Imaging Laboratory of Core Facility Center, Capital Medical University, Beijing, China

**Keywords:** tetramethylpyrazine, ischemic stroke, neurogenesis, angiogenesis, astrocytes, neurovascular unit

## Abstract

2,3,5,6-Tetramethylpyrazine (TMP) as an active ingredient extracted from a traditional Chinese herbal medicine *Ligusticum chuanxiong Hort*. has been proved to penetrate blood-brain barrier (BBB) and show neuroprotective effects on cerebral ischemia. However, whether TMP could regulate astrocytic reactivity to facilitate neurovascular restoration in the subacute ischemic stroke needs to be urgently verified. In this research, permanent occlusion of the middle cerebral artery (MCAO) model was conducted and TMP (10, 20, 40 mg/kg) was intraperitoneally administrated to rats once daily for 2 weeks. Neurological function was evaluated by motor deficit score (MDS). Magnetic resonance imaging (MRI) was implemented to analyze tissue injury and cerebral blood flow (CBF). Magnetic resonance angiography (MRA) was applied to exhibit vascular signals. Transmission electron microscopy (TEM) was performed to detect the neurovascular unit (NVU) ultrastructure. Haematoxylin and eosin (HE) staining was utilized to evaluate cerebral histopathological lesions. The neurogenesis, angiogenesis, A1/A2 reactivity, aquaporin 4 (AQP4) and connexin 43 (Cx43) of astrocytes were observed with immunofluorescent staining. Then FGF2/PI3K/AKT signals were measured by western blot. Findings revealed TMP ameliorated neurological functional recovery, preserved NVU integrity, and enhanced endogenous neurogenesis and angiogenesis of rats with subacute ischemia. Shifting A1 to A2 reactivity, suppressing excessive AQP4 and Cx43 expression of astrocytes, and activating FGF2/PI3K/AKT pathway might be potential mechanisms of promoting neurovascular restoration with TMP after ischemic stroke.

## 1. Introduction

Ischemic stroke is the primary cause of high disability and mortality globally. Following the neuroprotective agents failing in clinical trials, the focus of stroke treatment shifted from neuroprotection to preserving neurovascular unit (NVU; Zagrean et al., 2018). NVU is a functionally and structurally interdependent multicellular complex consisting of neurons, endothelia, astrocytes, microglia, smooth muscle cells, and pericytes (del Zoppo, 2010). The intricate contact of NVU exhibits in a coordinated manner to control blood-brain barrier (BBB), regulate cerebral perfusion as well as maintain microenvironment homeostasis (Becerra-Calixto and Cardona-Gomez, 2017; Becerra-Calixto et al., 2018).

As the vital components of NVU, astrocytes surround neurons and vessels *via* the specialized endfeet to maintain BBB integrity (Chen and Swanson, 2003; Bambrick et al., 2004). Importantly, astrocytes are appreciated for their specialized functions in regulating blood flow *via* regulating NVU activity. Enriched on astrocytic endfeet, aquaporin 4 (AQP4) is highly selective for water transport and sustains neuronal excitability and BBB permeability (Kong et al., 2008; Vandebroek and Yasui, 2020), while connexin 43 (Cx43)-mediated gap junction communication modulates neurotransmission, and impacts neuronal activity and synaptic plasticity (Liu et al., 2012; Belousov et al., 2017; Wang et al., 2019). This suggests that astrocytes are key to supporting and regulating NVU in the central nervous system (CNS).

When challenged with ischemia and hypoxia stimulation, astrocytes were induced by two distinct phenotypes termed A1 and A2 respectively (Liddelow and Barres, 2017). A1 astrocytes secrete neurotoxin and pro-inflammatory factors like IL-1α, TNF-α, and IFN-γ (Li K. et al., 2019). By contrast, A2 astrocytes possess the anti-inflammatory property and initiate endogenous neurorestorative processes including angiogenesis and neurogenesis (Liu and Chopp, 2016; Liddelow and Barres, 2017; Michinaga and Koyama, 2019). Studies have also documented that fibroblast growth factor 2 (FGF2) derived from A2 astrocytes plays a key role in stimulating cell proliferation and differentiation *via* the phosphoinositide 3-kinase/Akt (PI3K/Akt) signaling pathway (Xing et al., 2015; Zhao et al., 2019). Hence, inducing the transformation of astrocytes into the anti-inflammatory A2 subtype, possessing an excellent role in NVU preservation and neurovascular remodeling after ischemic stroke.

2,3,5,6-Tetramethylpyrazine (TMP) has been widely used to treat cardiovascular and cerebrovascular diseases (Tan et al., 2015). Pharmacological researches reported that TMP treatment could protect BBB integrity, promote newborn neuronal migration, alleviate cerebral infarction, and eventually improve neurological function after ischemic stroke (Xiao et al., 2010; Cai et al., 2014; Tan et al., 2015; Gong et al., 2019). In particular, TMP inhibits astrocytic activation in ischemic penumbra of acute ischemia/reperfusion rats (Liao et al., 2004). On these grounds, we hypothesize that TMP acts on reactive astrocytes, which might be critical for neurovascular remodeling and functional recovery after stroke.

In this study, magnetic resonance imaging (MRI) and histological detections were performed to explore TMP’s effectivities on neurovascular restoration. Furthermore, we detected the expressions of AQP4 and Cx43, astrocytic A1/A2 reactivity, and FGF2/PI3K/AKT pathway to develop in-depth knowledge of the possible repair mechanisms underlying TMP treatment after ischemic stroke.

## 2. Materials and methods

### 2.1. Animals and drugs

Seventy-six male Sprague Dawley rats weighing 280–320 g (aged 2 months) were purchased from Vital River Laboratory Animal Technology Co., Ltd. and raised in specific pathogen-free animal research center in Capital Medical University [SYXK (jing) 2018-0003]. Animal care and experimental protocols were followed with the guidelines set by the National Institute of Health Guide for the Care and Use of Laboratory Animals, and approved by Capital Medical University Animal Ethics Committee (Permit Number: AEEI-2018-052). TMP hydrochloride injection (HPLC > 98%) was purchased from Harbin Medisan Pharmaceutical Co., Ltd. (Lot No. 090923A, Harbin, Heilongjiang, China).

### 2.2. Permanent ischemic model and experimental groups

Focal cerebral ischemia was induced by intraluminal occlusion of the right middle cerebral artery (MCAO) in line with our previous recordation (Feng et al., 2022). The successful model identified that rats could circle or walk to the left were involved in the present experiment (Yu et al., 2013). Sixty MCAO rats were randomly divided into the model group (*n* = 18), TMP 40 mg/kg group (*n* = 14), TMP 20 mg/kg group (*n* = 14), and TMP 10 mg/kg group (*n* = 14). Sham-operated rats were grouped into the sham group (*n* = 10). TMP dissolved in saline was intraperitoneally injected into rats at 4 h after MCAO and once daily for successive 2 weeks. Rats in the sham and model groups were injected with the same volume of saline (1 ml/kg/day).

### 2.3. Neurological function evaluation

The motor deficit score (MDS) was conducted and the specific tests included observation of spontaneous ipsilateral circling, contralateral hindlimb retraction, beam walking ability, and bilateral forepaw grasp of ischemic rats (Altumbabic et al., 1998; Tamakoshi et al., 2014). It was measured on the 3rd, 7th, 10th, and 14th days after MCAO by observers blinded to experimental groups (*n* = 10). MDS was graded on a scale of 0-12, and the higher score represented the more severe deficit of neurological function after ischemic injury.

### 2.4. MRI scanning and analysis

MRI experiments were conducted with a 7.0 T animal MRI scanner (Bruker, PharmaScan, Germany) on the 15th day post-treated with TMP. During MRI scanning, rats (*n* = 6) were anesthetized with isoflurane (5% for induction and 2% for maintenance) and settled in a feedback-controlled system to keep the rectal temperature at 37 ± 0.5°C.

T2 relaxometry mapping was utilized to analyze tissue injury (Zhang et al., 2016). Regions of interest (ROIs) including infarction, periinfarct cortex, and striatum were sketched according to the rat brain atlases (Schober, 1986). T2 values of ROIs were obtained according to the previous recordation (Feng et al., 2022). Relative T2 value (rT2) was calculated as the relative value of ipsilateral and contralateral T2 value.

Arterial spin labeling (ASL) could detect CBF (Zhang et al., 2016, 2019). The relative CBF value (rCBF) of the periinfarct cortex or striatum was the ipsilateral CBF value divided by the contralateral CBF value (Yang et al., 2015). Magnetic resonance angiography (MRA) was conducted to determine the Willis circle and collateral circulation (Hoksbergen et al., 2003). The coronal multiplanar reformation (MPR) and maximal intensity projection (MIP) of MRA were acquired with Paravision version 5.1 software (Naiganawa, 2015). The bilateral signal intensities of the anterior cerebral artery (ACA), anterior communicating artery (AComA), anterior azygos cerebral artery (azACA), middle cerebral artery (MCA), internal carotid artery (ICA), posterior cerebral artery (PCA) and basilar artery (BA) were respectively acquired with ImageJ software (Kara et al., 2012; Zhan et al., 2020).

### 2.5. Tissue processing

After MRI scanning, rats were anesthetized for histopathological evaluation (*n* = 4) and ultrastructural observation (*n* = 2) as the previous method (Zhan et al., 2020). Haematoxylin and eosin (HE) staining was conducted to examine the cerebral histopathological lesion. Three nonoverlapping microscopic areas were randomly sampled from the periinfarct cortex and striatum in each rat (Li M. Z. et al., 2019), then nerve cells were calculated and presented by the average cell number/mm^2^.

### 2.6. Transmission electron microscopy analysis

The ultrastructural changes in NVU in periinfarct cortex were examined with H7700 transmission electron microscopy (TEM; Hitachi, Tokyo, Japan; Zhan et al., 2020). An average of 25 microvessels were captured from each rat to assess the ultrastructures of microvessels and perivascular astrocytes. The number of endothelial vesicles was calculated and presented by vesicles/vascular lumen circumference (μm). Pericyte coverage was calculated as the vascular lumen circumference covered by a pericyte/vascular lumen circumference (Haley and Lawrence, 2017). The thickness of basement membrane was the average length of four line segments on microvessels (Nahirney et al., 2016; Zhan et al., 2020). The swelling ratio of astrocytic endfeet was calculated as the swollen area of endfeet/vascular lumen area (Nahirney et al., 2016). The injury severity of mitochondria in astrocytic endfeet was scored as followed: Score zero, intact mitochondrial structure; Score one, disappeared matrix granules; Score two, swollen mitochondria appearance; Score three, disintegrated mitochondrial cristae; Score four, destroyed or disappeared mitochondrial membranes (Hou et al., 2016; Feng et al., 2022).

Additionally, the alleviation of neuronal damage was evaluated thus an average of 20 neurons were captured from each rat. The injury degree of neurons was scored according to the standard (Shi et al., 2014): Score zero, normal structure; Score one, slightly injured, distended endoplasmic reticulum and swollen mitochondria; Score two, mildly injured, swollen organelles and dented karyolemma; Score three, moderately injured, arisen cytoplasmic vacuoles, shrunken cell nucleus, transparent cytoplasm; Score four, severely injured, pyknotic cell membranes and karyolemma; Score 5, near-death, disrupting cell membranes, ruptured karyolemma and disintegrating organelles.

### 2.7. Immunofluorescence staining

Immunofluorescence staining was conducted as previous methods (Zarruk et al., 2018). Neurogenesis was visualized with Ki67 (label proliferative cells)/MAP-2 (label mature neurons). Angiogenesis was visualized with Ki67/CD31 (label endothelial cells), and CD31/NG2 (label pericytes; Zhan et al., 2020). A1/A2 reactive astrocytes were respectively visualized with complement C3 (C3)/glial fibrillary acidic protein (GFAP, label activated astrocytes) and S100 calcium binding protein A 10 (S100A10)/GFAP (Miyamoto et al., 2020). Functional proteins of astrocytes were visualized with, AQP4/GFAP (Liang et al., 2016), Cx43/GFAP (Une et al., 2021), and FGF2/GFAP (Shi et al., 2018).

Briefly, the sections were incubated overnight at 4°C in the following primary antibodies: rabbit anti-GFAP (1:1,000; GeneTex, GTX108711), anti-AQP4 (1:100; Santa Cruz Biotechnology, sc-20812), anti-Cx43 (1:100; Abcam, ab66151), anti-C3 (1:400; Abcam, ab200999), anti-Ki67 (1:100; GeneTex, GTX16667), anti-CD31 (1:200; Abcam, ab28364), anti-NG2 (1:200; Abcam, ab50009); mouse anti-GFAP (1:600; Millipore, MAB360), anti-S100A10 (1:500; Cell Signaling Technology, 5529s), anti-MAP-2 (1:100; Abcam, ab11267) and anti-FGF2 (1:500; Santa Cruz Biotechnology, sc-365106). After the sections were washed, primary antibodies were detected with secondary antibodies including goat anti-mouse IgG (H + L)-TRITC (SouthernBiotech, 1031-03), goat anti-mouse IgG (H + L)-FITC (SouthernBiotech, 1036-02), goat anti-rabbit IgG-FITC (SouthernBiotech, 4030-02) and goat anti-rabbit IgG-TRITC (SouthernBiotech, 4030-03), followed by counterstained DNA in the nuclei with 4’,6-diamidino-2-phenylindole (DAPI). Three fields of the periinfarct cortex and striatum were randomly selected to obtain the intensity of GFAP^+^/AQP4^+^, GFAP^+^/Cx43^+^ and FGF2^+^, and count the number of Ki67^+^, Ki67^+^/MAP-2^+^, Ki67^+^/CD31^+^, CD31^+^/NG2^+^, GFAP^+^/C3^+^, GFAP^+^/S100A10^+^, and GFAP^+^/FGF2^+^ cells. Data were expressed by the average intensity or cells/mm^2^ (Haldorsen et al., 2014). Analyses above were performed blindly to experimental groups (*n* = 4).

### 2.8. Western blot analysis

Rats (*n* = 4) without undergoing MRI experiments were deeply anesthetized, and periinfarct cortices were quickly dissected. Protein quantification and western blot analyses were followed with previous procedures (Zhan et al., 2020). Proteins were separated by SDS-PAGE and probed with the corresponding primary antibodies: rabbit anti-AQP4 (1:20,000; Santa Cruz Biotechnology, sc-20812), anti-Cx43 (1:10,000; Abcam, ab66151), anti-VEGF (1:8,000; Abcam, ab4051), anti-angiogenin (Ang)-1 (1:2,000; Millipore, AB10516), anti-Ang-2 (1:2,000; Abcam, ab125692), anti-GFAP (1:150,000; GeneTex, GTX108711), anti-C3 (1:16,000; Abcam, ab200999), anti-S100A10 (1:1,000; Immunoway, YT4198), anti-p-PI3K p85 (Tyr458)/p55 (Tyr199; 1:5,000; Cell Signaling Technology, 4228), anti-PI3K (1:20,000; Cell Signaling Technology, 4257), anti-p-AKT(Ser473; 1:20,000; Cell Signaling Technology, 4060), anti-AKT (1:40,000; Cell Signaling Technology, 4691); mouse anti-FGF2 (1:2,000; Santa Cruz Biotechnology, sc-365106), anti-STAT3 (1:10,000; Cell Signaling Technology, 9139) and anti-GAPDH (1:160,000; GeneTex, GTX627408). Intensities of proteins were acquired quantitatively with ImageJ system.

### 2.9. Statistical analysis

Data were presented as mean ± standard error of the mean (SEM) and analyzed with SPSS 26.0 software. Data of the MDS, T2 relaxometry mapping, ASL, and immunofluorescence staining were analyzed by Student’s *t*-test. Other experimental data were analyzed by one-way analysis of variance followed by Bonferroni’s *post-hoc* test. The correlation between AQP4/Cx43 and C3/S100A10 was analyzed with Pearson linear regression. Significance was set at *P < 0.05 and **P < 0.01.

## 3. Results

### 3.1. TMP ameliorated tissue injury and neurological function in ischemic rats

**Figure 1:**
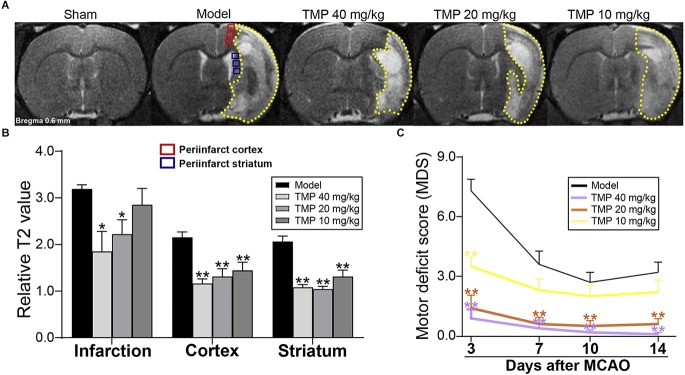
TMP improved tissue injury and neurological function of MCAO rats. **(A)** Typical T2 relaxometry maps showed the tissue injury of rats (*n* = 6). Quantitative analysis of **(B)** relative T2 value (rT2) of infarction (yellow dotted lines), periinfarct cortex (red box), and striatum (blue box) at the 14th day after MCAO, and **(C)** motor deficit score (MDS) at the 3rd, 7th, 10th, and 14th day after MCAO (*n* = 10). *P < 0.05, **P < 0.01 vs. Model group.

T2 relaxometry mapping was performed to evaluate the structural changes in cerebral tissues (Figure 1A). Quantitative results showed that rT2 values of infarction in MCAO rats were significantly decreased by TMP (20, 40 mg/kg; *p* < 0.05), and TMP (10, 20, 40 mg/kg) downregulated rT2 values in the periinfarct cortex and striatum compared with the model group (*p* < 0.01), revealing that TMP ameliorated tissue injury after stroke (Figure 1B).

Neurological function evaluation showed significantly decreased MDS in TMP (20, 40 mg/kg)-treated rats at the 3rd, 7th, 10th, and 14th days after ischemia (*p* < 0.01), and TMP (10 mg/kg) downregulated the MDS at the 3rd day after MCAO compared with the model group (*p* < 0.01; Figure 1C), suggesting TMP promoted the motor outcomes of ischemic rats.

### 3.2. TMP improved cerebral perfusion and collateral circulation establishment in ischemic rats

**Figure 2:**
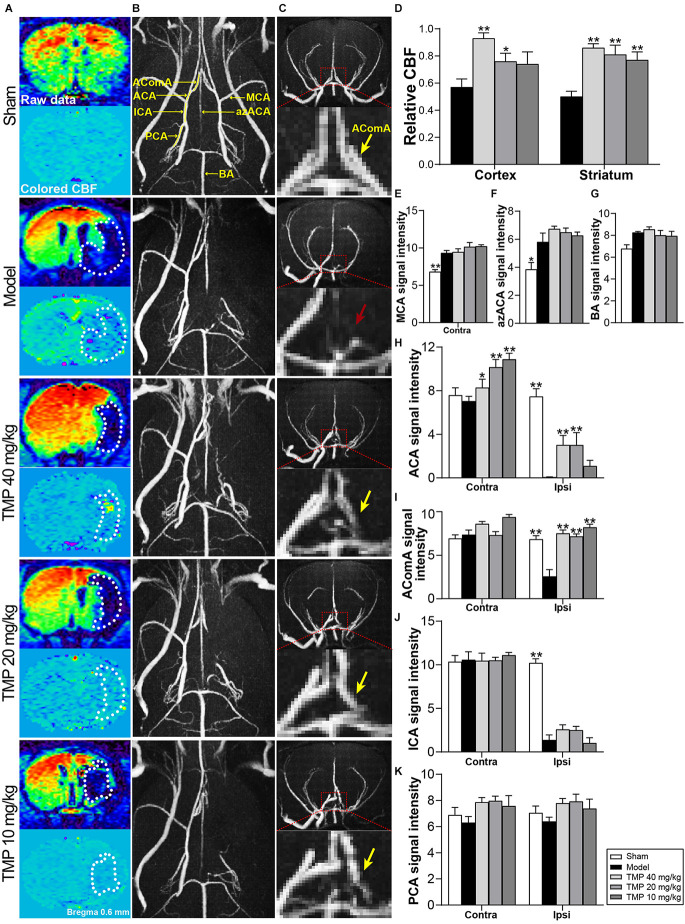
TMP improved cerebral perfusion and enhanced vascular signals of MCAO rats. **(A)** ASL and colored CBF images showed cerebral perfusion (*n* = 6). The infarctions were marked with white dotted lines. **(B)** Axial and **(C)** coronal MRA images showed cerebral vascular signals (*n* = 6). Signals in ipsilateral MCA regions were obviously absent in the Model group. AComA areas were magnified in coronal images (red dotted boxes). The disappeared AComA signal (red arrow) was indicated in the Model group and it reappeared in TMP groups (yellow arrows). **(D)** Quantitative analysis of rCBF in the periinfarct cortex and striatum, and signal intensity of **(E)** MCA, **(F)** azACA, **(G)** BA, **(H)** ACA, **(I)** AComA, **(J)** ICA, and** (K)** PCA. Abbreviations: MCA, middle cerebral artery; ACA, anterior cerebral artery; AComA, anterior communicating artery; ICA, internal carotid artery; PCA, posterior cerebral artery; azACA, anterior azygos cerebral artery; BA, basilar artery; Contra, contralateral; Ipsi, ipsilateral. *P < 0.05, **P < 0.01 vs. Model group.

CBF of ischemic rats was quantified with MRI-ASL (Figure 2A). *Post-hoc* comparisons revealed that TMP (20, 40 mg/kg) efficaciously upregulated rCBF in periinfarct cortex and striatum (*p* < 0.05 or *p* < 0.01), and rCBF of periinfarct striatum in TMP (10 mg/kg) group was improved in comparison with model rats (*p* < 0.01; Figure 2D).

MRA was applied to assess the morphology and signals of intracranial vessels (Figures 2B,C). Compared with sham rats, model rats showed stronger signal intensities of ipsilateral azACA and contralateral MCA while weaker signal intensities of ipsilateral ICA, ACA, and AComA in rats (*p* < 0.05 or *p* < 0.01). TMP (10, 20, 40 mg/kg) treatment obviously increased the signal intensities of contralateral ACA and ipsilateral AComA in comparison with the model group (*p* < 0.05 or* p* < 0.01), besides, TMP (20, 40 mg/kg) upregulated the ipsilateral ACA signal intensity in MCAO rats (*p* < 0.01; Figures 2E–K). The above results suggested that TMP improved cerebral perfusion mainly by establishing collateral circulation of the anterior arterial system in ischemic rats.

### 3.3. TMP enhanced endogenous neurogenesis in ischemic rats

**Figure 3:**
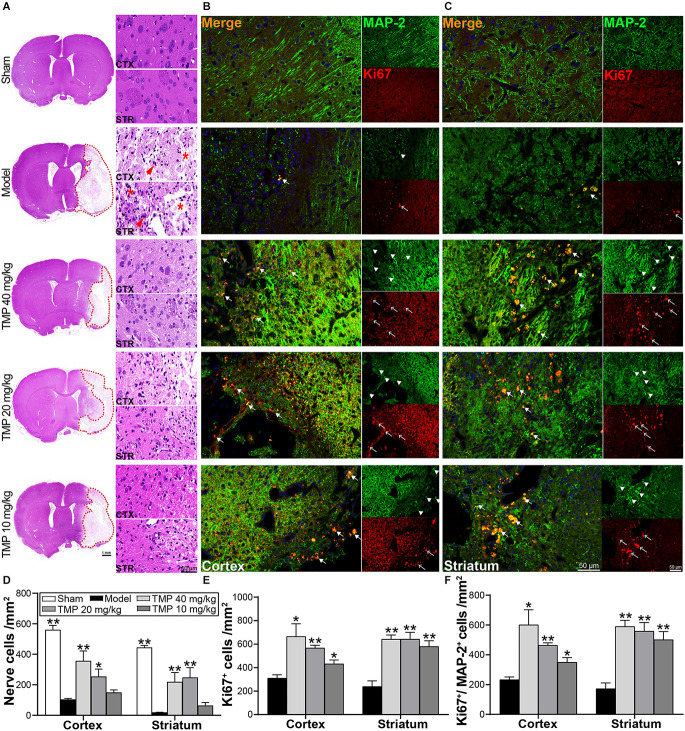
TMP improved endogenous neurogenesis of MCAO rats. **(A)** HE staining photographs showed nerve cells in the periinfarct cortex (CTX) and striatum (STR; *n* = 4). The infarctions were represented with red dotted lines. Abnormal nerve cells (red arrows) and disintegrated tissues (*) were indicated in the Model group. **(B,C)** Immunofluorescence images showed co-localization of MAP-2-stained mature neurons (green, arrowheads) and Ki67-stained proliferative cells (red, empty arrows) in peri-infarct cortex and striatum (*n* = 4). Merge (orange, arrows) indicated Ki67/MAP-2 co-labeling cells. DAPI (blue) indicated cell nuclei. Quantification of **(D)** nerve cell density, **(E)** Ki67^+^ cells, and** (F)** Ki67^+^/MAP-2^+^ cells in the periinfarct cortex and striatum of each group. *P < 0.05, **P < 0.01 vs. Model group.

HE staining exhibited the nerve cells in the perilesional cortex and striatum appeared with necrosis and pyknosis in model rats, and cell density was sharply decreased compared to sham rats (*p* < 0.01; Figure 3A). Whereas TMP (20, 40 mg/kg) treatment markedly improved nerve cell density in corresponding areas (*p* < 0.05 or *p* < 0.01; Figure 3D), proving TMP preserved nerve cells and alleviated histopathological injury after stroke.

Ki67 and MAP-2 in immunofluorescence staining exhibited neurogenesis in ischemic rats (Figures 3B,C). Compared with the model group, TMP (10, 20, 40 mg/kg) distinctly improved Ki67^+^ and Ki67^+^/MAP-2^+^ cells in the periinfarct cortex and striatum when (*p* < 0.05 or *p* < 0.01; Figures 3E,F), suggesting that TMP promoted cell proliferation and induced neuronal differentiation to peri-lesions of ischemic rats.

### 3.4. TMP boosted endogenous angiogenesis in ischemic rats

**Figure 4:**
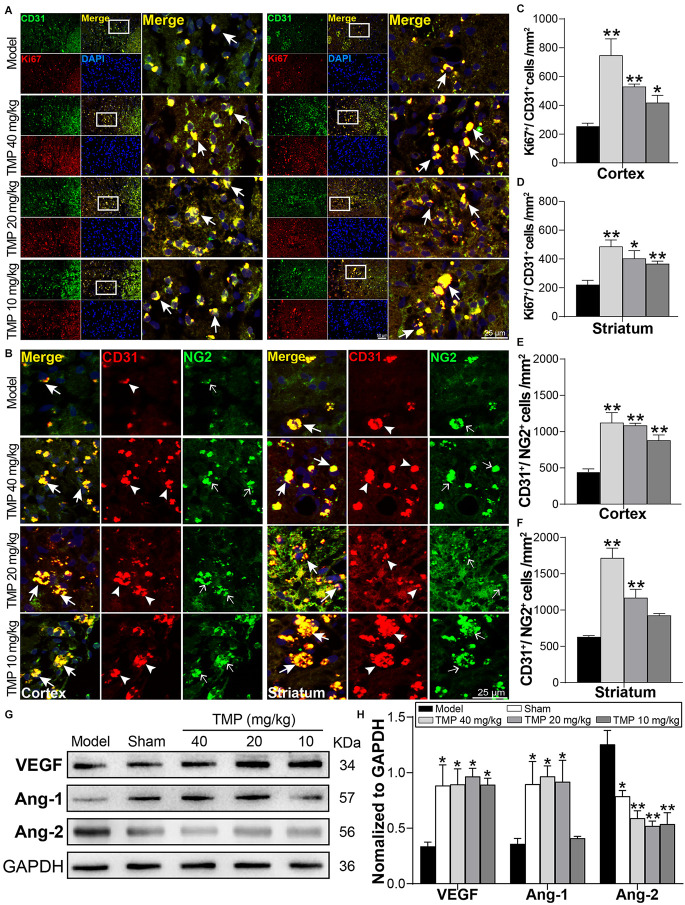
TMP boosted endogenous angiogenesis of MCAO rats. **(A)** Immunofluorescence images showed co-localization of CD31-stained endothelial cells (green) and Ki67-stained proliferative cells (red) in peri-infarct cortex and striatum (*n* = 4). Merge (yellow) indicated Ki67/CD31 co-labeling cells. **(B)** Immunofluorescence images showed co-localization of CD31 (red, arrowheads) and NG2-stained pericytes (green, empty arrows) in peri-infarct cortex and striatum (*n* = 4). Merge (yellow, arrows) indicated CD31/NG2 co-labeling cells. DAPI (blue) indicated cell nuclei. **(C–F)** Quantification of Ki67^+^/CD31^+^ and CD31^+^/NG2^+^ cells in peri-infarct cortex and striatum of Model and TMP groups. **(G,H)** Western blot analysis for VEGF, Ang-1, and Ang-2 in the periinfarct cortex (*n* = 4). *P < 0.05, **P < 0.01 vs. Model group.

To evaluate the impact of TMP on angiogenesis in the periinfarctions, Ki67/CD31 and CD31/NG2 were included to immunofluorescence staining (Figures 4A,B). By comparison with model rats, TMP (10, 20, 40 mg/kg) obviously upregulated the number of Ki67^+^/CD31^+^ cells in peri-infarct cortex and striatum (*p* < 0.05 or *p* < 0.01), demonstrating that TMP promoted cell proliferation and endothelia differentiation post-ischemia. Additionally, CD31^+^/NG2^+^ cells in peri-infarct cortex and striatum were respectively increased by TMP (10, 20, 40 mg/kg) and TMP (20, 40 mg/kg) compared with the model group (*p* < 0.01), which revealed the formation of new vessels was stimulated with TMP’s treatment (Figures 4C–F).

Furthermore, Western blot results showed the angiogenesis-associated factors VEGF and Ang-1 levels were obviously lower while Ang-2 was higher in the model ischemic cortex compared to the sham group (*p* < 0.05). Remarkably, in comparison with model rats, TMP (10, 20, 40 mg/kg) significantly improved VEGF and attenuated Ang-2 expression (*p* < 0.05 or *p* < 0.01), and TMP (20, 40 mg/kg) improved the level of Ang-1 of ischemic rats (*p* < 0.05; Figures 4G,H). Taken together, TMP facilitated the formation of vascular cells and regulated the expressions of VEGF, Ang-1, and Ang-2 in periinfarctions, thus improving the angiogenesis post-ischemic injury.

### 3.5. TMP ameliorated ultrastructural injuries of NVU in ischemic rats

**Figure 5:**
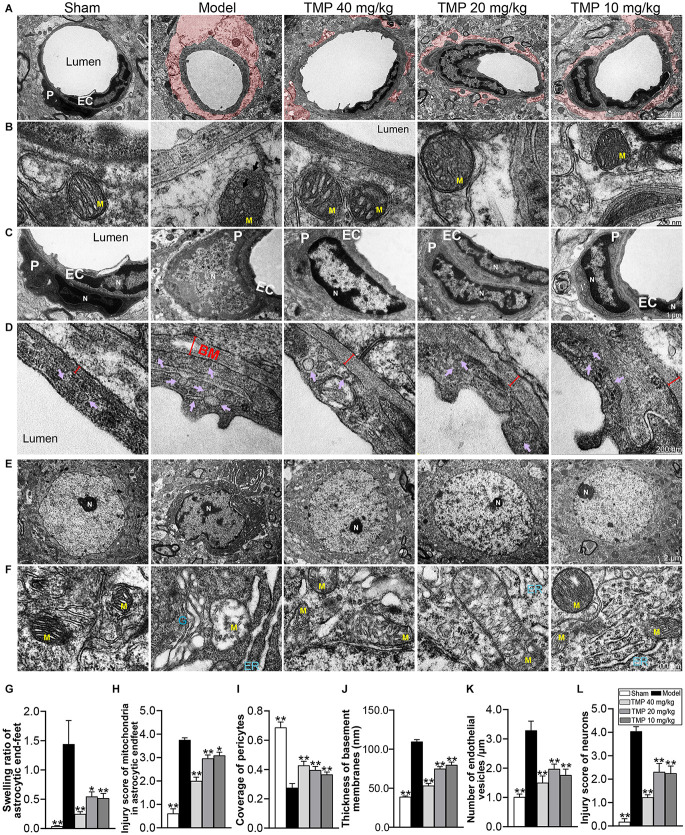
TMP ameliorated the ultrastructural injuries of NVU in perilesional cortex of MCAO rats. **(A)** Typical microvascular images showed the morphology of endothelial cells (EC), pericytes (P), and circumvascular astrocytes (*n* = 2). The swollen astrocytic endfeet were shaded in red. **(B)** Mitochondria (M) in astrocytic endfeet appeared the disintegrating cristae in the Model group. **(C)** Pericytes in microvessels were swollen and disintegrated from endothelial cells, and cell nuclei (N) showed heterogeneity, thickened nuclear membranes and irregular boundaries in the Model group. **(D)** Basement membranes (BM, red double-headed arrows) were thickened and caveolae-like vesicles (purple arrows) in endothelial cells increased in the Model group. **(E)** Typical neuronal images showed that circumvascular neurons and **(F)** circumambient organelle including mitochondria (M), endoplasmic reticulum (ER), and Golgi apparatus (G) were badly damaged in Model group. Quantification of **(G)** swelling ratio of astrocytic endfeet, **(H)** injury score of mitochondria in astrocytic endfeet, **(I)** coverage of pericytes to endothelial cells, **(J)** basement membrane thickness, **(K)** number of vesicles in endothelial cells/μm and **(L)** injury score of neurons. *P < 0.05, **P < 0.01 vs. Model group.

TEM was further conducted to analyze the ultrastructural injuries of NVU. TEM images of model microvessels exhibited the microvascular lumens in irregular shapes, endothelia with numerous caveolae-like vesicles and pericytes separated from endothelial cells. Besides, the thickened BM and swollen perivascular astrocytes were obvious in the model ischemic cortex (Figures 5A–D). Quantification revealed that compared with the sham group, the endothelial vesicles, BM thickness, swelling ratio of perivascular astrocytic endfeet, and injury score of mitochondria in endfeet were significantly increased, while the coverage of pericyte was decreased in model rats (*p* < 0.05 or *p* < 0.01). After treatment with TMP (10, 20, 40 mg/kg), the corresponding microvascular measures were obviously overturned in comparison with the model group (*p* < 0.05 or *p* < 0.01; Figures 5G–K).

Next, TEM images of neurons showed the nuclear in irregular shape and heterochromatin conspicuously aggregated into dark clusters upon ischemia. Besides, the circumambient organelle such as mitochondria were swollen with disintegrating cristae, the endoplasmic reticulum formed stacks filled with dark materials, and the Golgi apparatus dilated at both terminals (Figures 5E,F). *Post-hoc* comparisons revealed that the neuron injury score was upregulated in model rats compared to sham rats (*p* < 0.01). In contrast, TMP (10, 20, 40 mg/kg) significantly down-regulated the neuronal injury score (*p* < 0.01) and protected the ultrastructure of organelle around neurons (Figure 5L). To sum up, TMP had the capacity of protecting the NVU integrity.

### 3.6. TMP inhibited AQP4 and Cx43 expressions in astrocytes in ischemic rats

**Figure 6:**
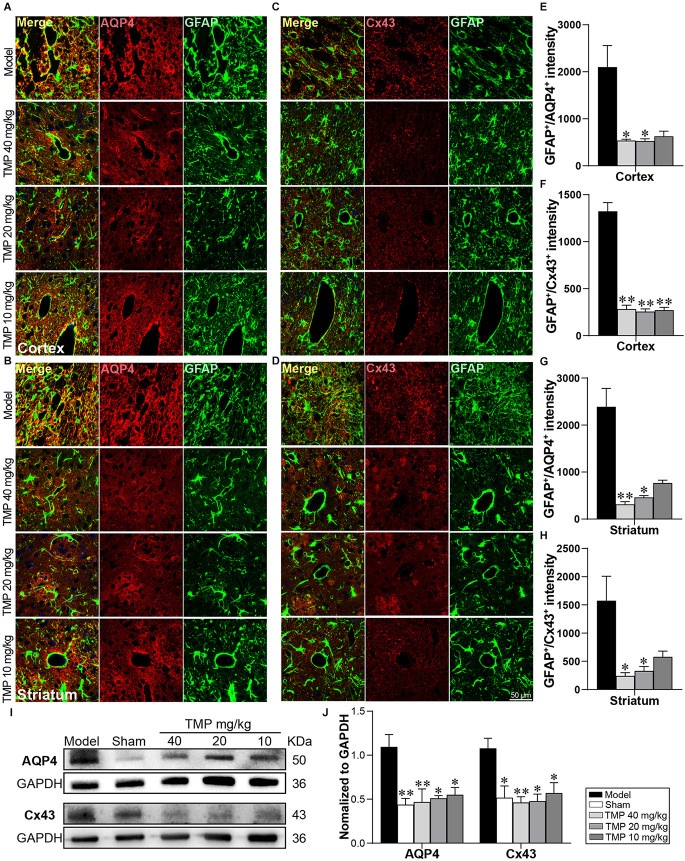
TMP inhibited the AQP4 and Cx43 expressions in astrocytes of MCAO rats. Immunofluorescence images showed co-localization of **(A,B)** GFAP (green) and AQP4 (red) and **(C,D)** GFAP (green) and Cx43 (red) in the periinfarct cortex and striatum (*n* = 4). Merge (yellow) indicated GFAP/AQP4 or GFAP/Cx43 co-labeling cells and DAPI (blue) indicated cell nuclei. **(E–H)** Quantification of GFAP^+^/AQP4^+^ and GFAP^+^/Cx43^+^ intensities in the periinfarct cortex and striatum of Model and TMP groups. **(I,J)** Western blot analysis for AQP4 and Cx43 in the periinfarct cortex (*n* = 4). *P < 0.05, **P < 0.01 vs. Model group.

Functional proteins of astrocytes AQP4 and Cx43 were respectively co-labeled with GFAP in immunofluorescence staining (Figures 6A–D). In comparison with model rats, TMP (20, 40 mg/kg) significantly decreased GFAP^+^/AQP4^+^ intensity in the periinfarct cortex and striatum (*p* < 0.05 or* p* < 0.01), and GFAP^+^/Cx43^+^ intensity in periinfarct striatum (*p* < 0.01). Additionally, TMP (10 mg/kg) reduced GFAP^+^/Cx43^+^ intensity in peri-infarct cortex (*p* < 0.01; Figures 6E–H). Western blot exhibited the expressions of AQP4 and Cx43 in the periinfarct cortex were increased in model rats compared to the sham group while downregulated by TMP (10, 20, 40 mg/kg; *p* < 0.05 or *p* < 0.01; Figures 6I,J), suggesting that TMP inhibited the overexpression of AQP4 and Cx43 in astrocytes.

### 3.7. TMP regulated astrocytic A1/A2 reactivity in ischemic rats

**Figure 7:**
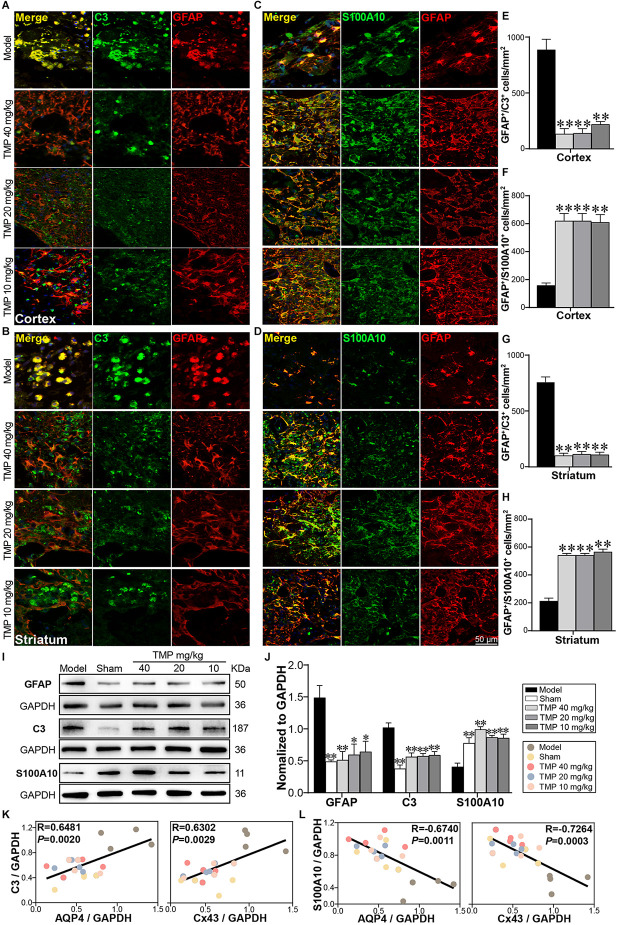
TMP regulated A1/A2 astrocytic reactivity of MCAO rats. Immunofluorescence images showed co-localization of **(A,B)** GFAP (red) and C3 (green) and** (C,D)** GFAP (red) and S100A10 (green) in the periinfarct cortex and striatum (*n* = 4). Merge (yellow) indicated GFAP/C3 or GFAP/S100A10 co-labeling cells. DAPI (blue) in merge pictures indicated cell nuclei. **(E–H)** Quantification of GFAP^+^/C3^+^ cells and GFAP^+^/S100A10^+^ cells in the periinfarct cortex and striatum of Model and TMP groups. **(I,J)** Western blot analysis for GFAP, C3 and S100A10 in the periinfarct cortex (*n* = 4). **(K,L)** Correlations of the relative expressions between astrocytic functional proteins (AQP4, Cx43) and reactive astrocytic markers (C3, S100A10) in the periinfarct cortex. *P < 0.05, **P < 0.01 vs. Model group.

A1 and A2 reactive astrocytes were marked with immunofluorescence staining (Figures 7A–D). Compared to model rats, TMP (10, 20, 40 mg/kg) distinctly reduced GFAP^+^/C3^+^ cells while increased GFAP^+^/S100A10^+^ cells in the periinfarct cortex and striatum (*p* < 0.01; Figures 7E–H). Western blot showed GFAP and C3 expressions were increased and S100A10 decreased apparently in the model ischemic cortex compared with the sham group (*p* < 0.01). In line with immunofluorescence results, TMP (10, 20, 40 mg/kg) significantly downregulated GFAP and C3 levels while upregulated S100A10 expression (*p* < 0.05 or *p* < 0.01; Figures 7I,J), revealing that TMP intervention suppressed A1 while promoted A2 reactivity of astrocytes in MCAO rats.

Furthermore, Pearson linear regression analysis demonstrated that AQP4 was distinctly in positive correlation with C3 (*R* = 0.6481, *p* < 0.01) and in negative correlation with S100A10 (*R* = −0.6740, *p* < 0.01). Moreover, Cx43 was strongly in positive correlation with C3 (*R* = 0.6302, *p* < 0.01), and in negative correlation with S100A10 (*R* = −0.7264, *p* < 0.001) in the ipsilateral cortex (Figures 7K,L). These results revealed the downregulation of AQP4 and Cx43 expressions was highly consistent with A2 polarization of astrocytes in ischemic rats post-treated with TMP.

### 3.8. TMP enhanced FGF2 expression in astrocytes and activated FGF2/PI3K/AKT pathway in ischemic rats

**Figure 8:**
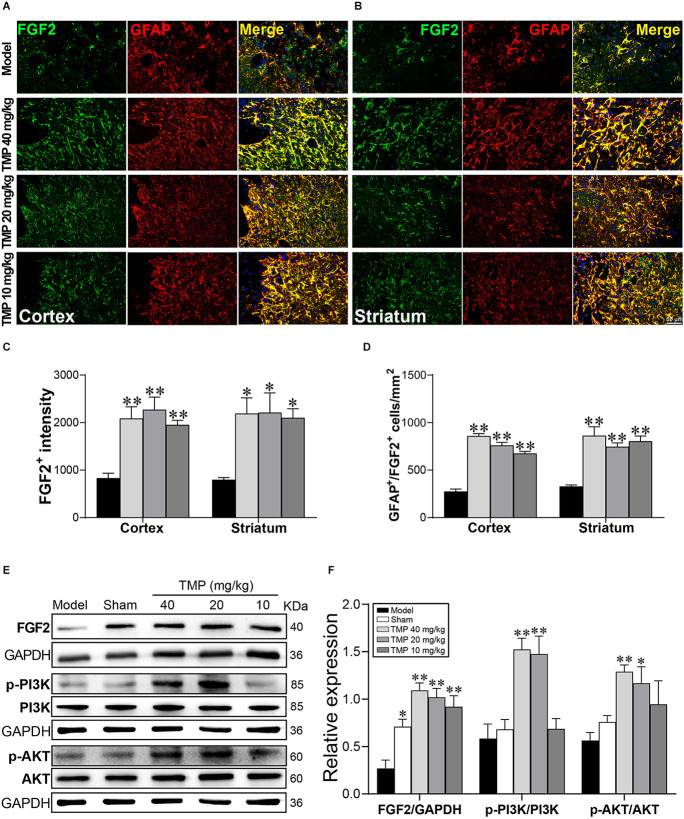
TMP increased FGF2 expression in astrocytes and activated PI3K/AKT/FGF2 pathway in MCAO rats. Immunofluorescence images showed co-localization of FGF2 (green) and GFAP (red)-stained astrocytes in **(A)** the periinfarct cortex and **(B)** striatum. Merge (yellow) indicated GFAP/FGF2 co-labeling cells and DAPI (blue) indicated cell nuclei. Quantification of **(C)** FGF2^+^ intensity and **(D)** GFAP^+^/FGF2^+^ cells in the periinfarct cortex and striatum of Model and TMP groups (*n* = 4).** (E,F)** Western blot analysis for FGF2, PI3K, and AKT in the periinfarct cortex (*n* = 4). *P < 0.05, **P < 0.01 vs. Model group.

To further validate the role of FGF2 in astrocytes, FGF2 was co-labeled with GFAP in immunofluorescence staining (Figures 8A,B). FGF2^+^ intensity and GFAP^+^/FGF2^+^ cells in the periinfarct cortex and striatum were obviously increased in TMP (10, 20, 40 mg/kg)-treated groups compared to the model group (*p* < 0.05 or *p* < 0.01; Figures 8C,D). TMP was confirmed to induce FGF2 release from astrocytes in ischemic rats.

FGF2 and downstream PI3K/AKT signals provide the neuroprotective effect against cerebral ischemia (Xing et al., 2015). Our western blot results showed the distinct lower level of FGF2 in the periinfarct cortex of model rats compared with the sham group (*p* < 0.05). TMP (20, 40 mg/kg) treatment significantly enhanced p-PI3K p85 (Tyr458)/p55 (Tyr199) and p-AKT (Ser473) levels, and TMP (10, 20, 40 mg/kg) upregulated the FGF2 expression (*p* < 0.05 or* p* < 0.01), revealing that TMP activated FGF2/PI3K/AKT pathway in ischemic rats (Figures 8E,F).

**Figure 9:**
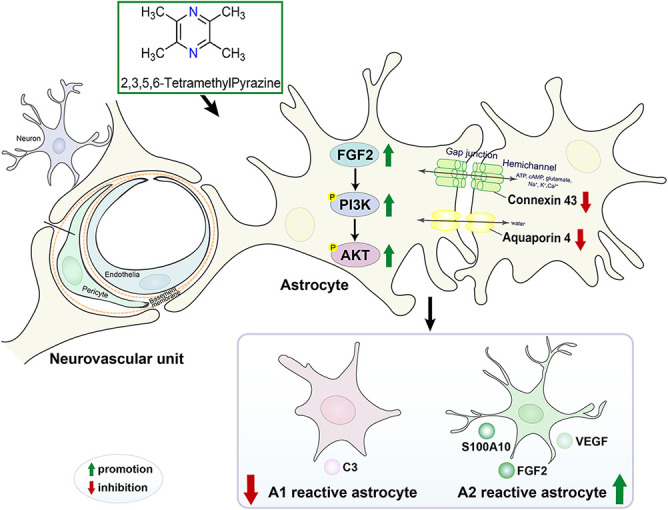
The mechanism diagram that TMP maintained NVU and reduced the neurological injury caused by cerebral ischemia.

In conclusion, the mechanism diagram that TMP maintained NVU and reduced the neurological injury caused by cerebral ischemia was exhibited in Figure 9.

## 4. Discussion

Based on the combination of noninvasive MRI and histopathological analysis, our current study proved that TMP ameliorated periinfarct tissue injury, cerebral perfusion, NVU ultrastructure, and eventually improved neurological function of subacute cerebral ischemic rats. Particularly, suppression of A1 and promotion to A2 reactivity are involved in the process of neurovascular restorative of TMP against ischemic injury.

In this study, multiparametric MRI was applied to delineate the tissue abnormality and neurovascular dysfunction. Down-regulated rT2 values obtained from T2 relaxometry mapping proved the tissue injuries in infarction, the periinfarct cortex, and striatum were relieved with TMP treatment. MRA imaging exhibited that TMP led to enhanced signal intensity of bilateral ACA and ipsilateral AComA in ischemic rats. AComA bridging bilateral ACA supports the anterior portion of Willis circle (Saleh et al., 2018), suggesting the augmented collateral flow through ACA to MCA territory of the ischemic side after TMP intervention. Specifically, MRI-ASL displayed poor hypoperfusion in the MCA territory of model rats, while TMP improved CBF of the periinfarct cortex and striatum. These MRI-based findings raised the interesting possibility that the beneficial effects of TMP toward collateral blood flow augmentation in the ipsilateral ischemic region after ischemic stroke.

Growing evidence has suggested a better collateral circulation might provide a favorable environment for angiogenesis and neurogenesis along the ischemic/hypoxic territory (Wei et al., 2018). Angiogenesis is defined as the formation of newborn microvessels by generated endothelia and activated pericytes (Shah et al., 2021). Notably, TMP induced angiogenesis as evidenced by the increased number of CD31 and NG2 proteoglycan. Angiogenesis is regulated by plenty of pro-angiogenic growth factors. According to our findings, TMP increased VEGF and Ang-1 expressions but decreased Ang-2 level, which was coincident with the boosted angiogenesis in the periinfarctions. VEGF mainly acts in the initial stage of microvascular network formation (Zhang et al., 2002; Zhao et al., 2010). In addition, Ang-1 and Ang-2 interacting with VEGF act on the late phase of angiogenesis after ischemia (Michalska-Jakubus et al., 2019). Ang-1 recruits pericytes and smooth muscle cells to stabilize BBB structure as well as improves cerebral perfusion (Lin et al., 2002; Michalska-Jakubus et al., 2019; Girolamo et al., 2022). Ang-2 is antagonistic to Ang-1 and disrupts vascular stability and induces pericytes to dissociate from endothelia thus resulting in BBB leakage (Kang et al., 2016; Rubio and Adamis, 2016). Amplified angiogenesis coupled with neurogenesis involves the process of neural progenitor cell proliferation and neuronal differentiation during brain tissue remodeling after stroke (Hatakeyama et al., 2020; Paro et al., 2022). We found TMP promoted neurogenesis as evidenced by increased Ki67^+^ and Ki67^+^/MAP-2^+^ cells in the ischemic cortex and striatum. Thus, the present study supported that TMP promoted endogenous neurovascular remodeling involving neurogenesis and angiogenesis following ischemic stroke.

Subsequently, we focused on the ultrastructural changes of NVU (e.g., neurons, endothelia, and perivascular astrocytes), which played a critical role in regulating CBF and energy metabolism (Kugler et al., 2021). TMP ameliorated the condensed chromatin in the nucleus and swollen organelles around neurons in peri-infarct cortex. Relieved neuronal injury is beneficial for cerebral infarct alleviation (Wilczynski, 2014; Tao-Cheng, 2018). After TMP treatment, caveolae and transcytotic vesicles in endothelia, basement membrane thickness, and pericyte coverage were all upregulated. Particularly, TMP functioned on alleviating the astrocytic endfeet swelling and mitochondria disruption inside of astrocytes. These encouraging changes of NVU ultrastructure strongly supported that TMP played an effective role in protecting NVU after stroke.

The underlying mechanisms of how TMP-induced neurovascular remodeling are yet to be elucidated. Specifically, astrocytes play a crucial role in protecting the neurovascular unit and facilitating angiogenesis and neurogenesis after stroke (Ding et al., 2021). It is worth noting that AQP4 and Cx43 predominately located on astrocytic endfeet are pivotal in neurovascular coupling (Isasi et al., 2014), and influencing the integrity of NVU (Cibelli et al., 2021). The abnormally upregulating AQP4 often correlates with BBB permeability after ischemia (Wolburg et al., 2009; Huang et al., 2018). AQP4 inhibition facilitated a reduction in astrocytic swelling thus reducing BBB disruption (Akdemir et al., 2014; Katada et al., 2014). Similarly, the sustained opening of Cx43 hemichannels and increased Cx43 expression after ischemia is likely to spread neurotoxic substances and apoptotic signals, which might potentially lead to NVU disruption (Xie et al., 2011; Davidson et al., 2013). And the inhibition of Cx43 hemichannel activity and Cx43 expression decreased the levels of glutamate and caspase-3, and then contributed to NVU cell survival after ischemia insults (Froger et al., 2010; Chen et al., 2019). In the present study, TMP reversed the overexpression of AQP4 and Cx43 located on astrocytes in periinfarctions of MCAO rats. Our findings illustrated AQP4 and Cx43 in TMP-mediated astrocytes following ischemic stroke, which brought new clues for exploring the mechanism of TMP-induced NVU protection and endogenous neurovascular remodeling.

It is worth noting that reactive astrocytes can be classified into A1 and A2 phenotypes, which provide neurotoxic and neuroprotective effects, respectively (Liddelow et al., 2017). Our research identified that TMP induced the transformation of astrocytes into A2 phenotype after stroke. Of greater interest, we found AQP4 and Cx43 levels were both strongly in positive correlation with C3 whereas in negative correlation with S100A10 expression, suggesting that AQP4 and Cx43 might be associated with polarization of astrocytes in cerebral ischemic rats. Previous studies have shown that Cx43 ablation was reported to be beneficial for A2 polarization of astrocytes in mice with multiple sclerosis (Une et al., 2021). Although far from being completely understood, the finding suggested that TMP induces the transformation of astrocytes into the neuroprotective A2 subtype at least in part, *via* regulating AQP4 and Cx43 after ischemic stroke.

It has been previously demonstrated that FGF2 released by A2 astrocytes play a crucial role in endogenous neurovascular remodeling (Chen et al., 1994). Particularly, several lines of evidence support that FGF2 activates PI3K/AKT as a classical pathway involved in neurogenesis and angiogenesis after cerebral ischemia (Chen et al., 2020; Zhan et al., 2020). In the present study, enhanced FGF2 expression in astrocytes was detected after TMP treatment. Consistent with the change of FGF2, a significantly activated PI3K/AKT pathway was measured in TMP-treated MCAO rats, suggesting that TMP-induced A2 astrocytic polarization and FGF2 release resulting in activation of PI3K/AKT pathway which might create a favorable microenvironment for neurovascular restoration, which also deserve future investigation.

More recently, we found TMP alleviated gray and white matter injury and enhanced axonal remodeling, contributing to the improvement of neurobehavioral function during the convalescence of cerebral ischemia (Feng et al., 2022). In the current study, our data provided evidence that TMP exhibited neurovascular restorative effects on stroke treatment *via* improving CBF, protecting NVU integrity, promoting endogenous neurogenesis and angiogenesis, inducing the A2 astrocytic polarization coupled with regulating AQP4 and Cx43 and activating FGF2/PI3K/AKT signals following ischemic stroke.

## Data availability statement

The original contributions presented in the study are included in the article/Supplementary materials, further inquiries can be directed to the corresponding author.

## Ethics statement

The animal study was reviewed and approved by Capital Medical University Animal Ethics Committee (Permit Number: AEEI-2018-052).

## Author contributions

X-fF performed experiments, analyzed data, and finished the manuscript. M-cL performed behavioral examination. Z-yL prepared samples. M-zL performed MRI examination. YL collected MRI data. Y-mZ collected behavioral data. J-fL prepared for MRI examination. LW provided experimental suggestions. HZ programmed the whole work and modified the final manuscript. All authors contributed to the article and approved the submitted version.

## Abbreviations

TMP: 2,3,5,6-Tetramethylpyrazine
BBB: blood-brain barrier
NVU: neurovascular unit
MRI: magnetic resonance imaging
AQP4: aquaporin 4
Cx43: connexin 43
MCAO: middle cerebral artery; MDS, motor deficit score
ASL: arterial spin labeling
MRA: magnetic resonance angiography
CBF: cerebral blood flow
TEM: transmission electron microscopy
HE: haematoxylin and eosin
VEGF: vascular endothelial growth factor
Ang-1: angiopoietin-1
FGF2: fibroblast growth factor 2
ACA: anterior cerebral artery
AComA: anterior communicating artery
azACA: anterior azygos cerebral artery
MCA: middle cerebral artery
ICA: internal carotid artery
PCA: posterior cerebral artery
BA: basilar artery.
