# Supplementary material for: Tetramethylpyrazine promotes stroke recovery by inducing the restoration of neurovascular unit and transformation of A1/A2 reactive astrocytes
Source: Front Cell Neurosci. 2023 Mar 27;17:1125412. doi: 10.3389/fncel.2023.1125412 (PMC10083399; doi:10.3389/fncel.2023.1125412)
Supplement: Supplementary file 2 [file Table_2.DOCX]

**Supplementary Figures**

| No. | Proteins | Original images of western blot | KDa |
| --- | --- | --- | --- |
| 1 | VEGF | 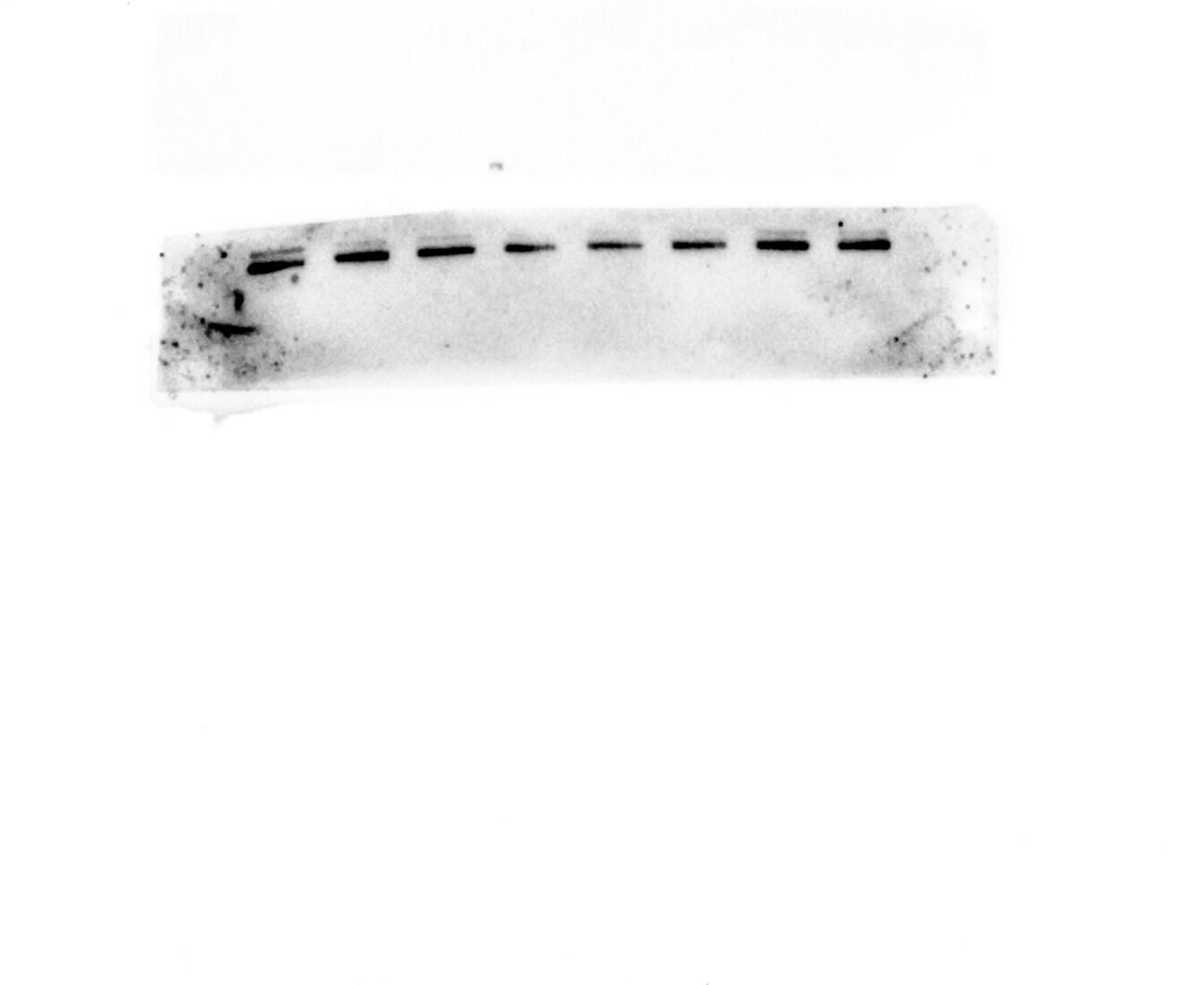 | 34 |
|  | GAPDH | 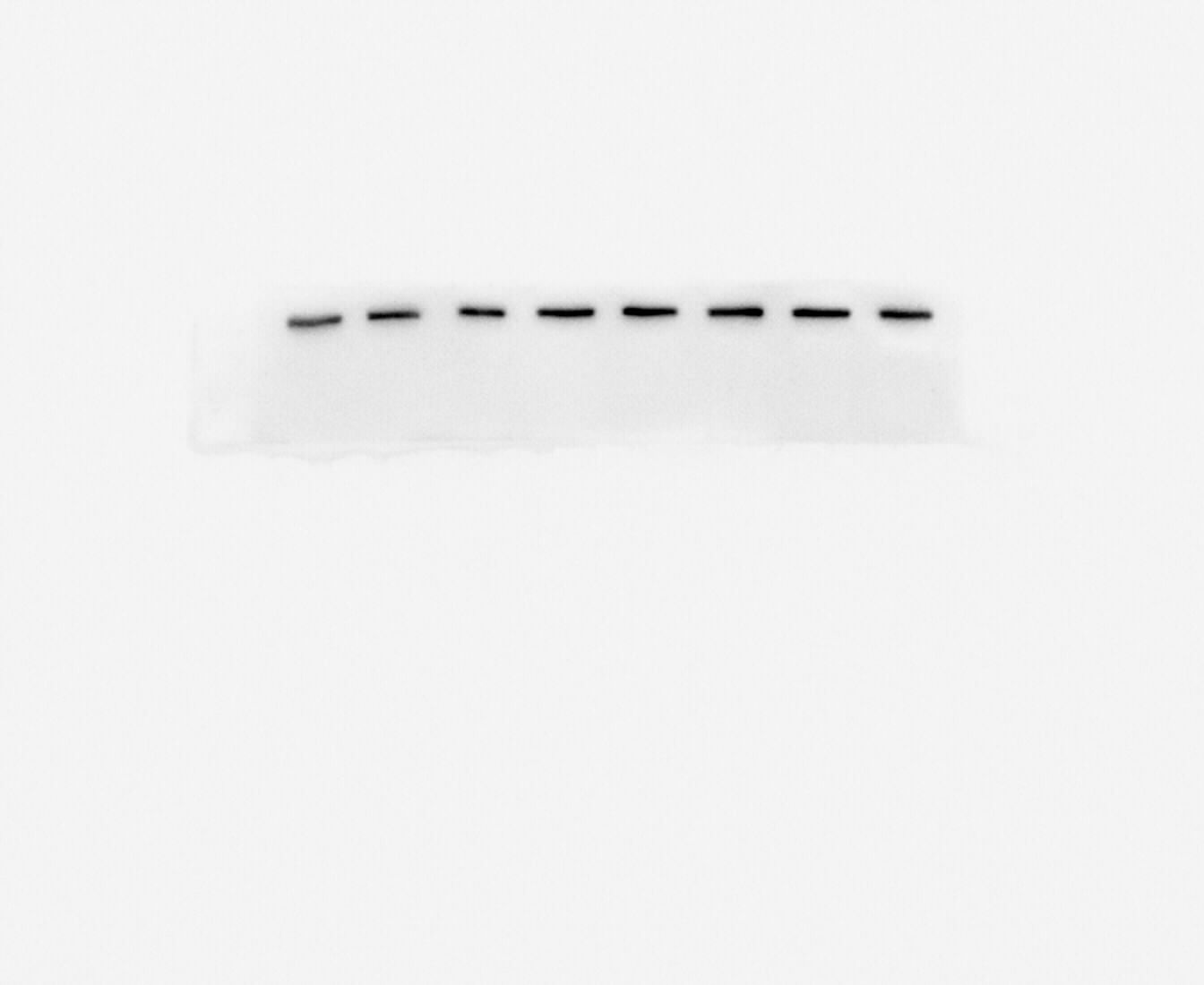 | 36 |
| 2 | Ang-1 | 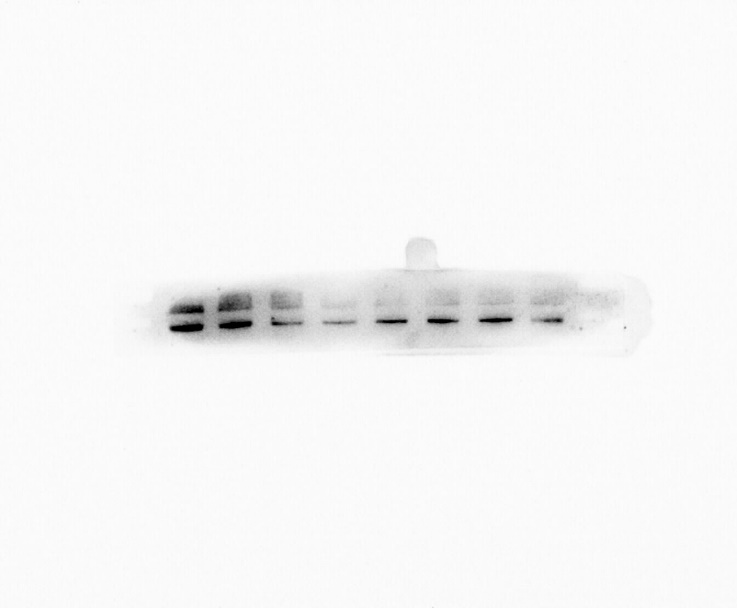 | 57 |
|  | GAPDH | 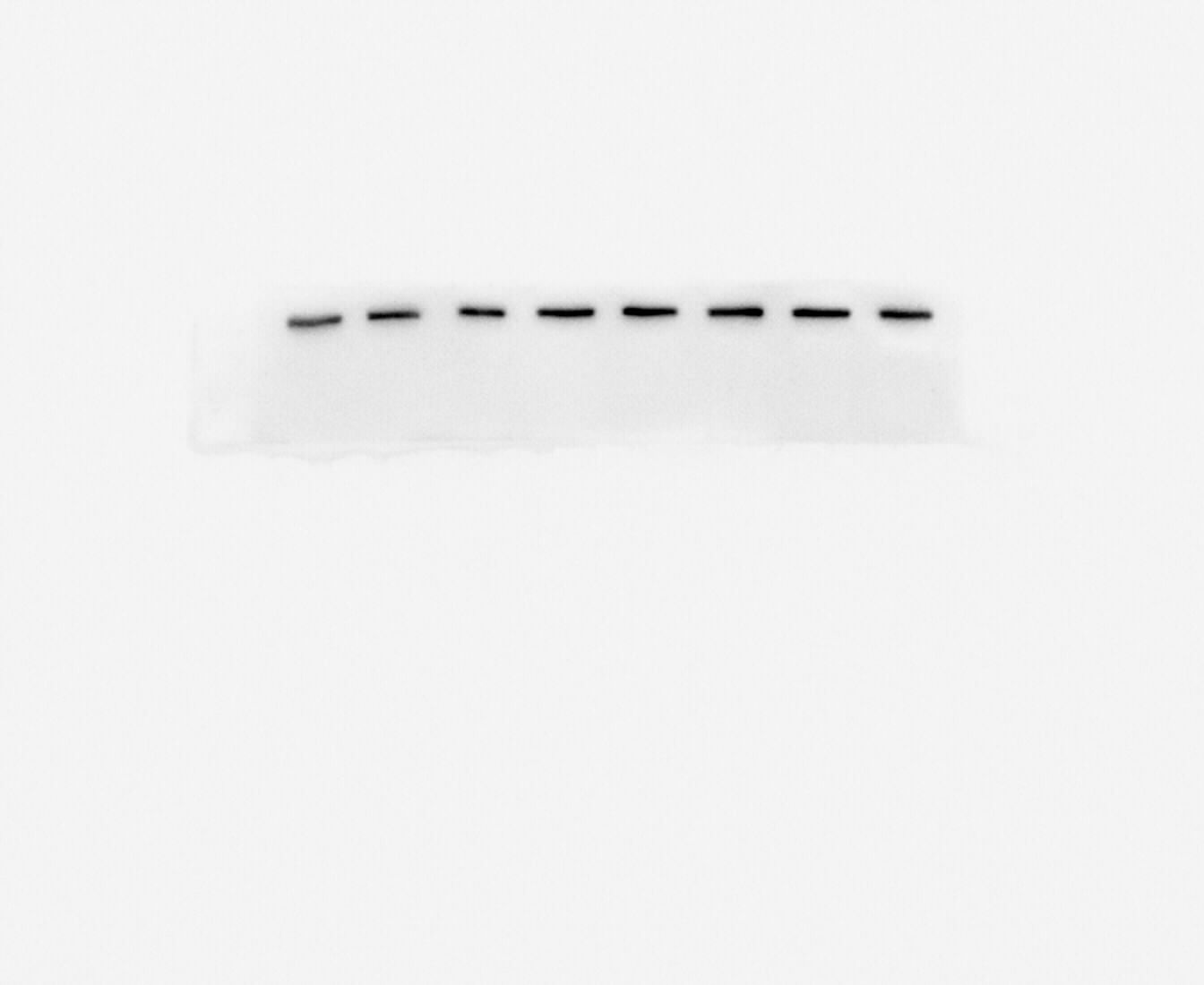 | 36 |
| 3 | Ang-2 | 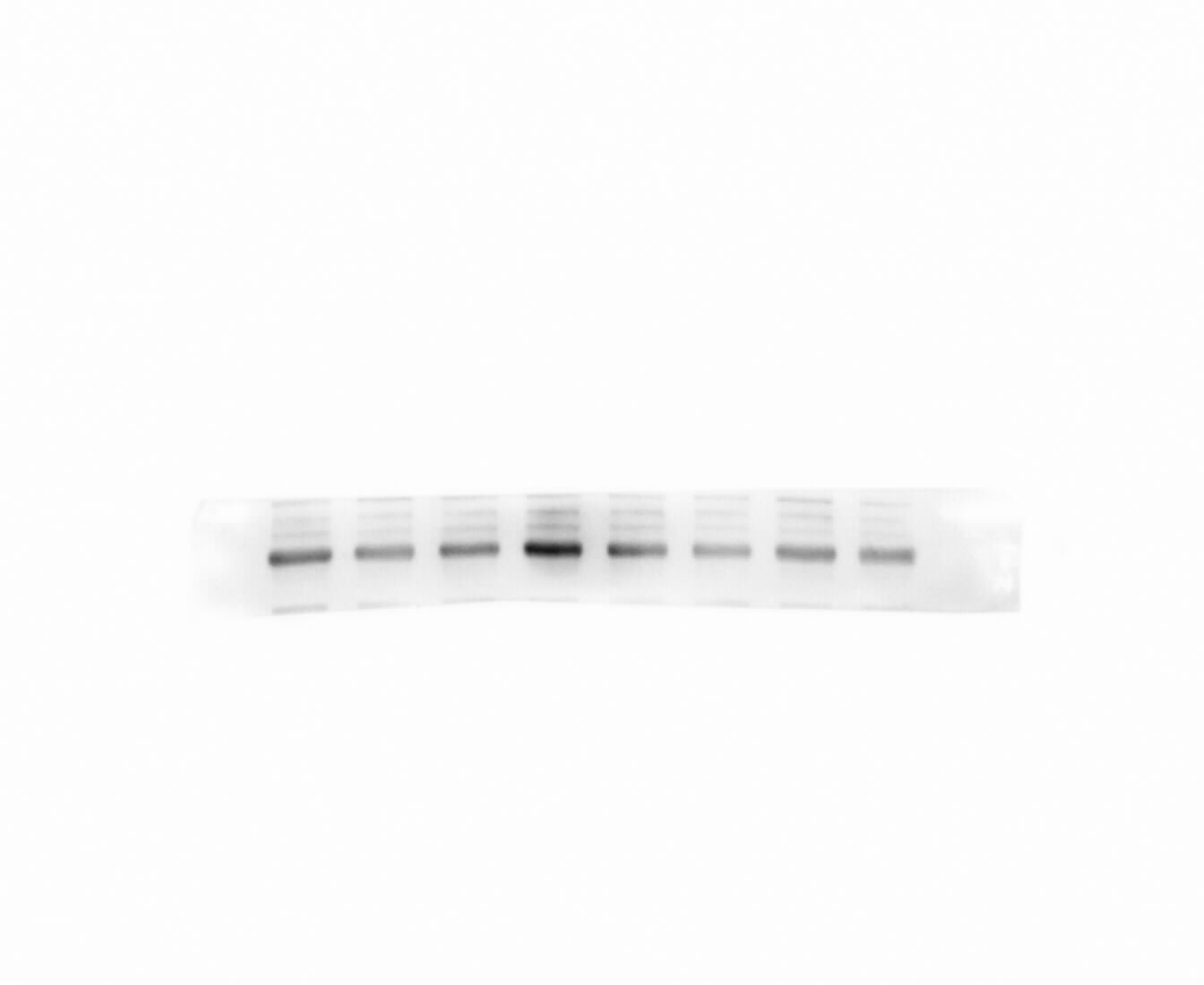 | 56 |
|  | GAPDH | 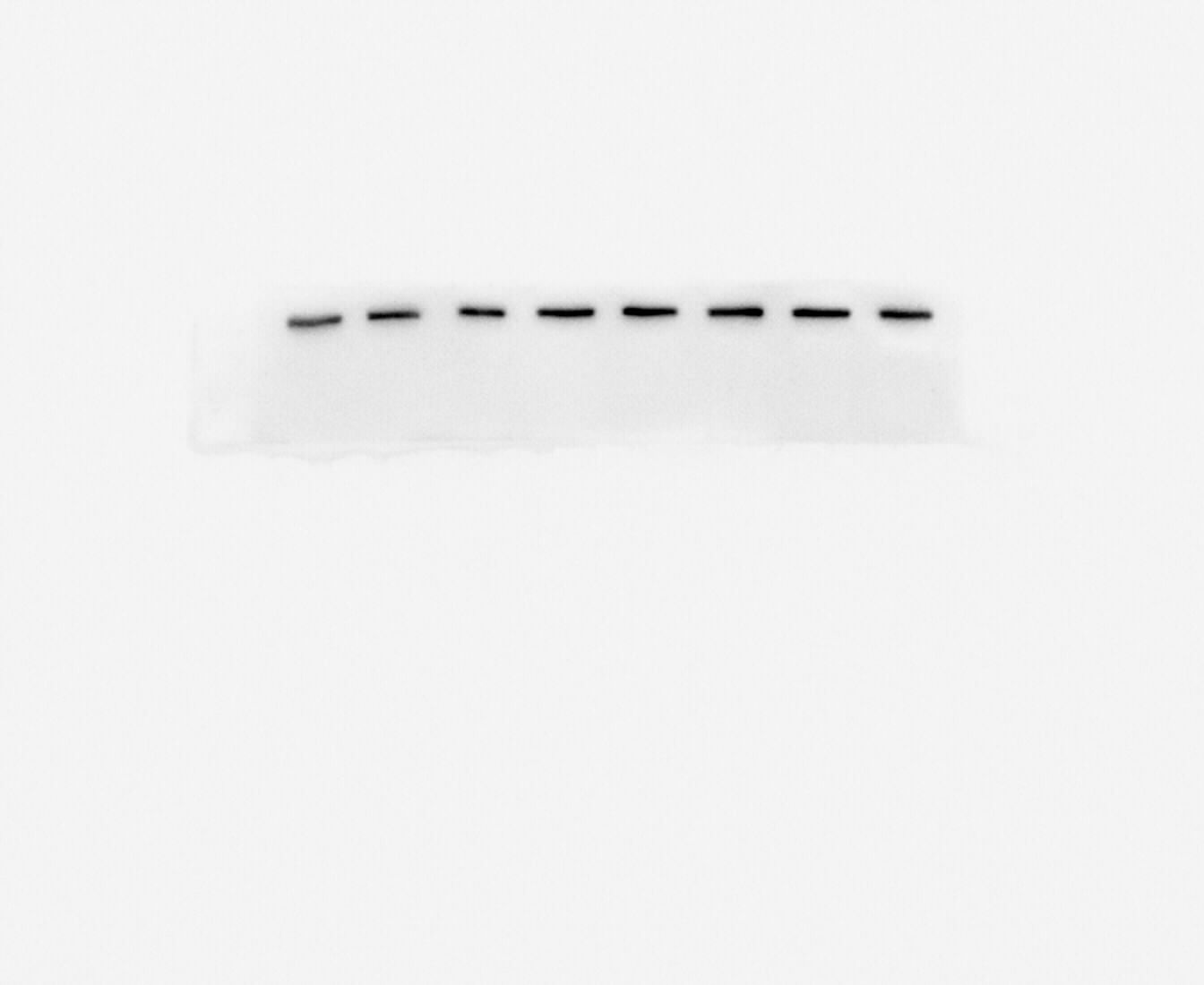 | 36 |
| 4 | AQP4 | 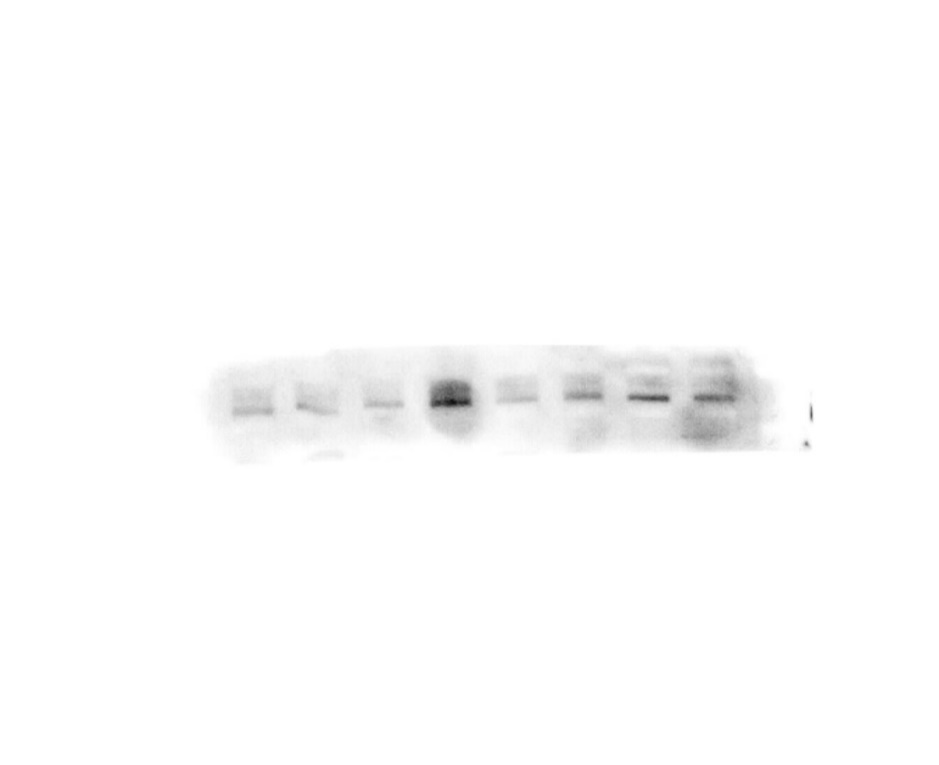 | 50 |
|  | GAPDH | 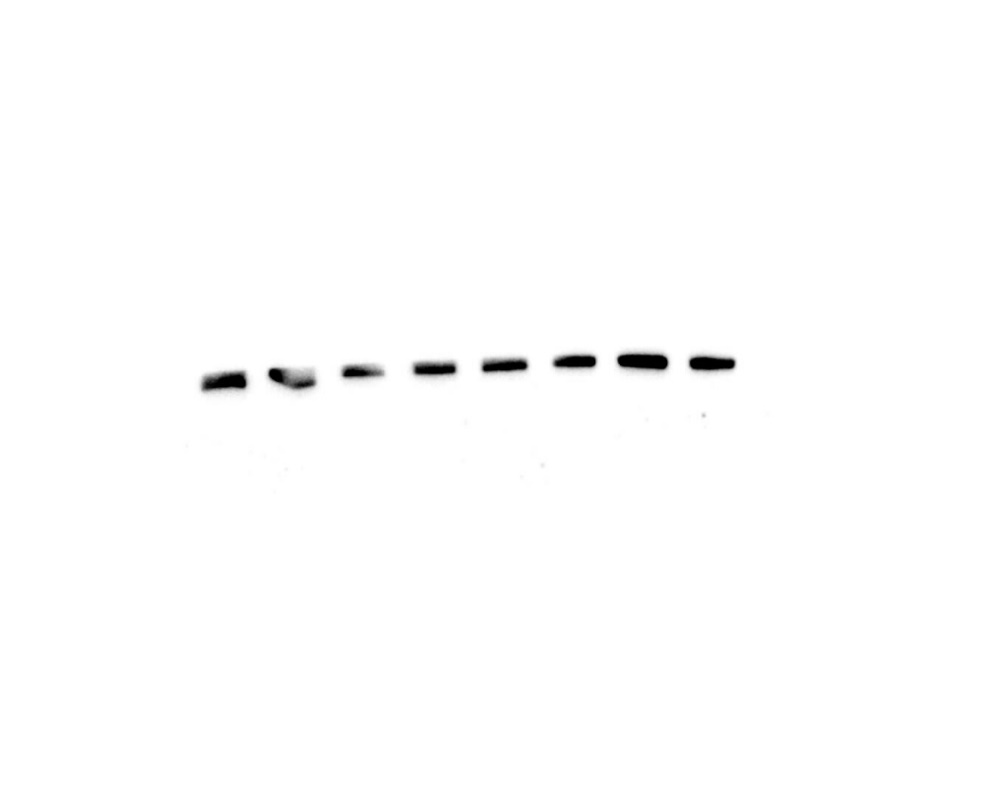 | 36 |
| 5 | Cx43 | 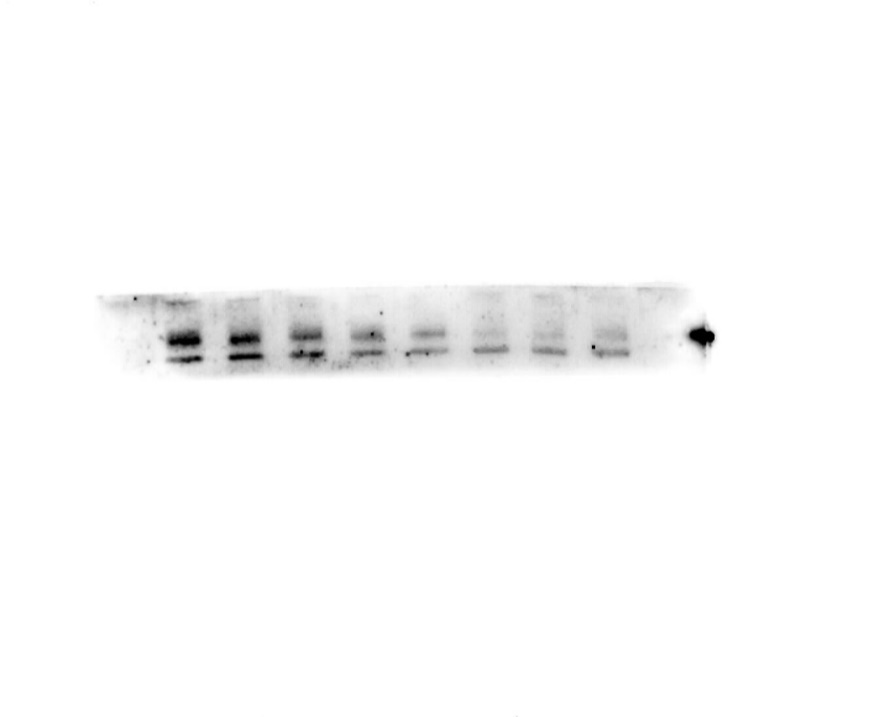 | 43 |
|  | GAPDH | 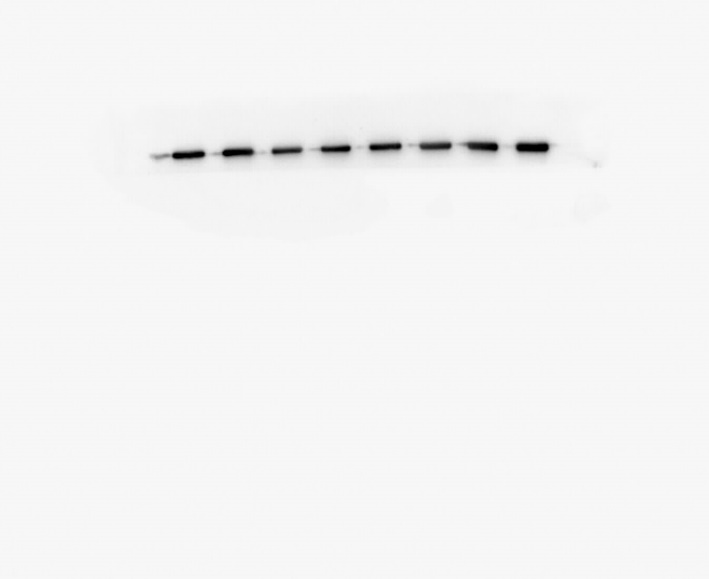 | 36 |
| 6 | GFAP | 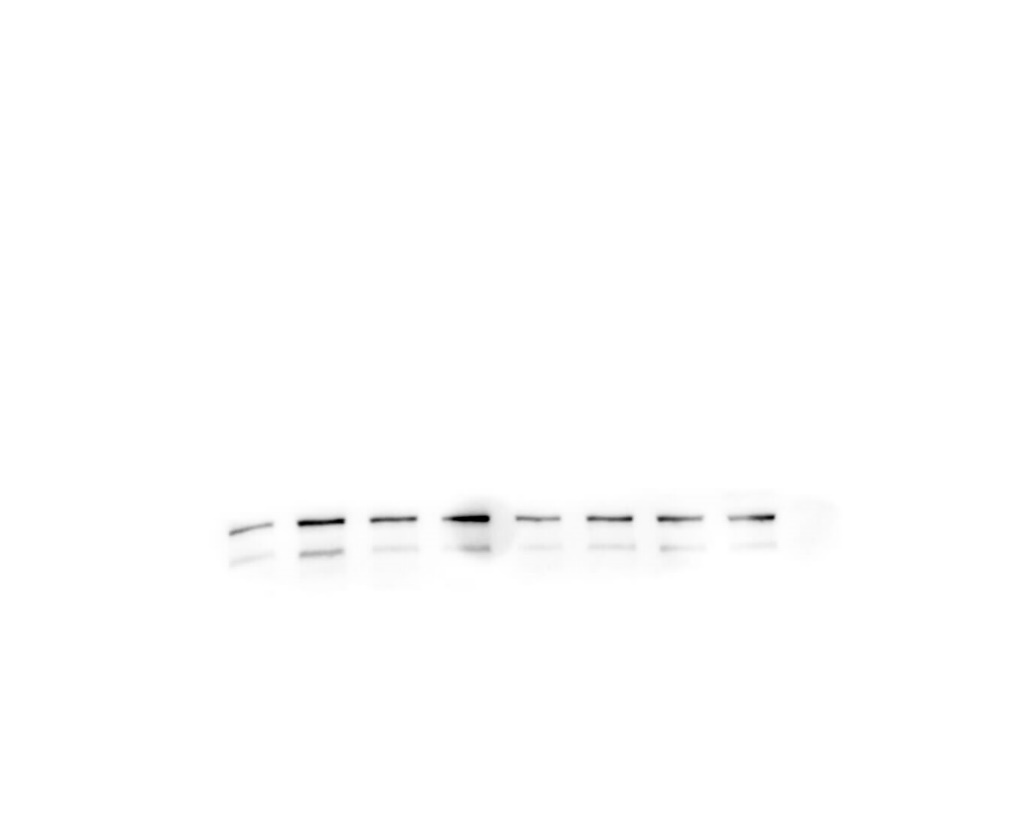 | 50 |
|  | GAPDH | 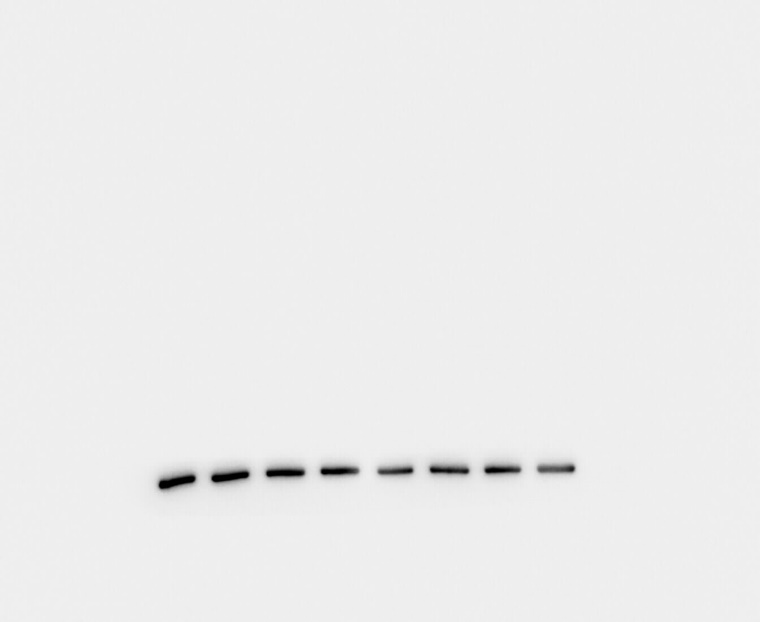 | 36 |
| 7 | C3 | 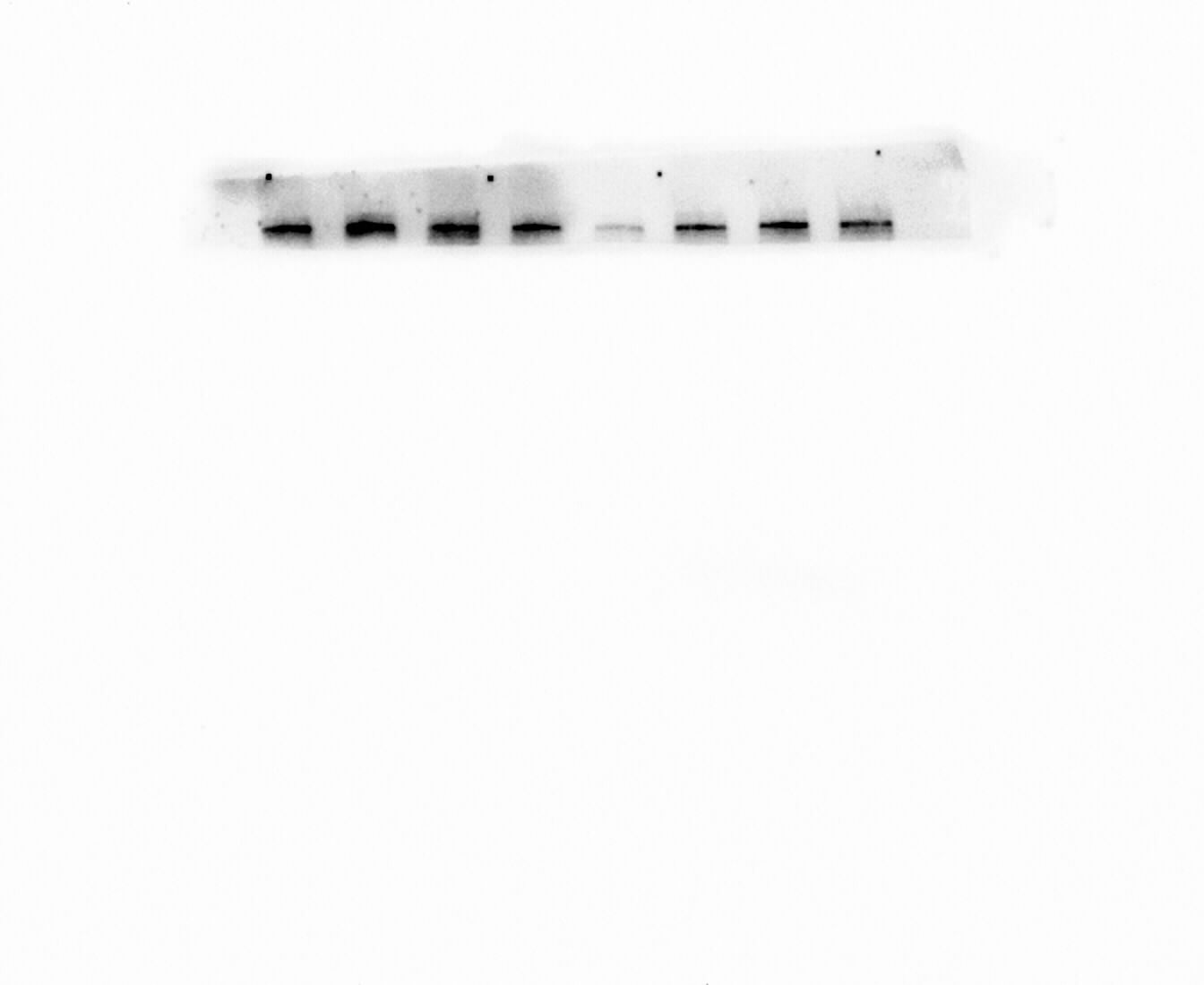 | 187 |
|  | GAPDH | 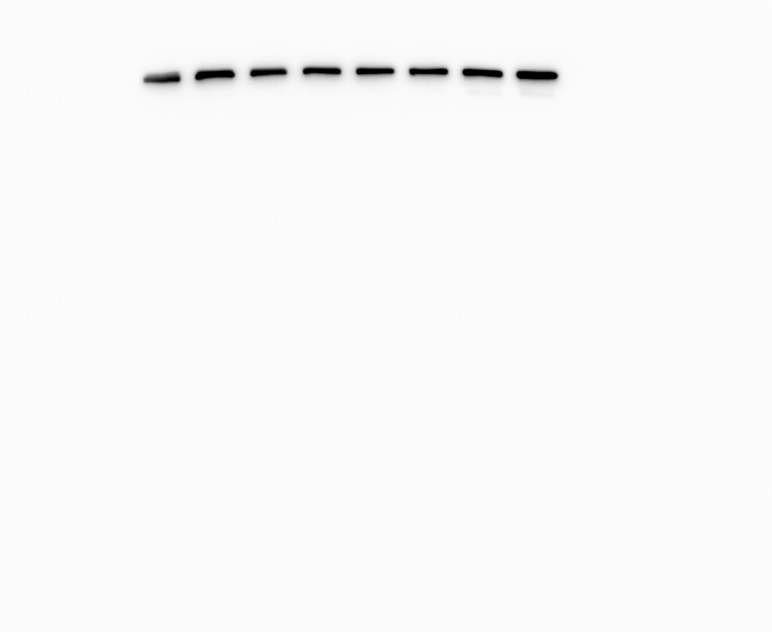 | 36 |
| 8 | S100A10 | 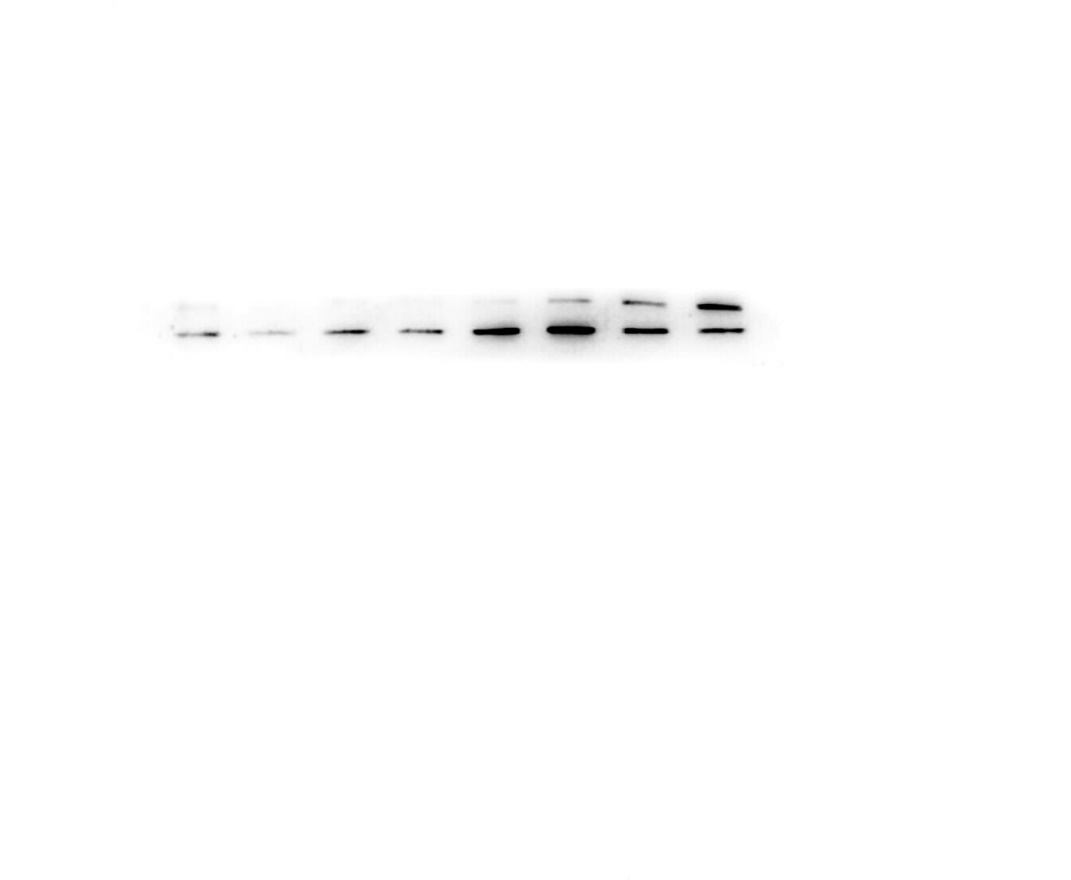 | 11 |
|  | GAPDH | 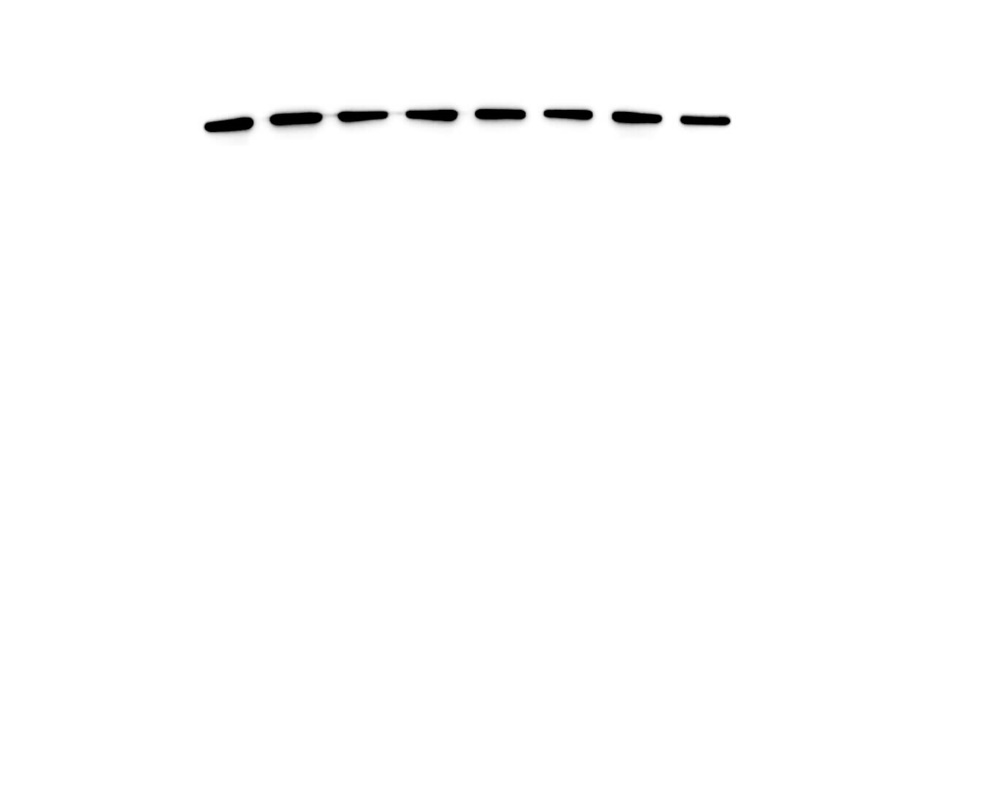 | 36 |
| 9 | FGF2 | 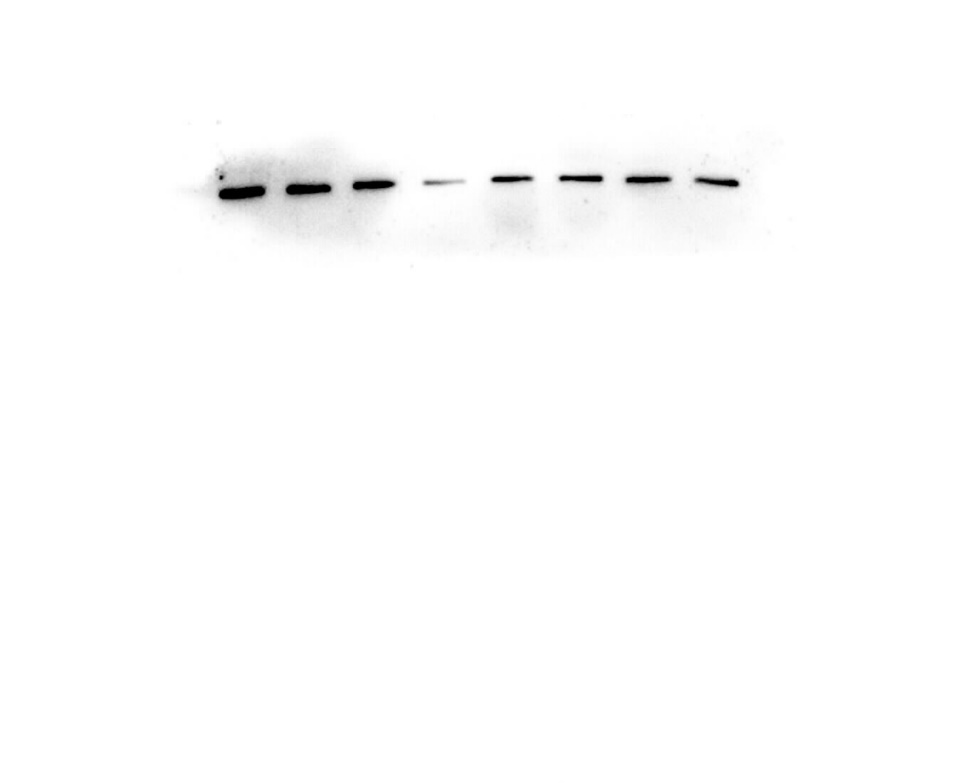 | 40 |
|  | GAPDH | 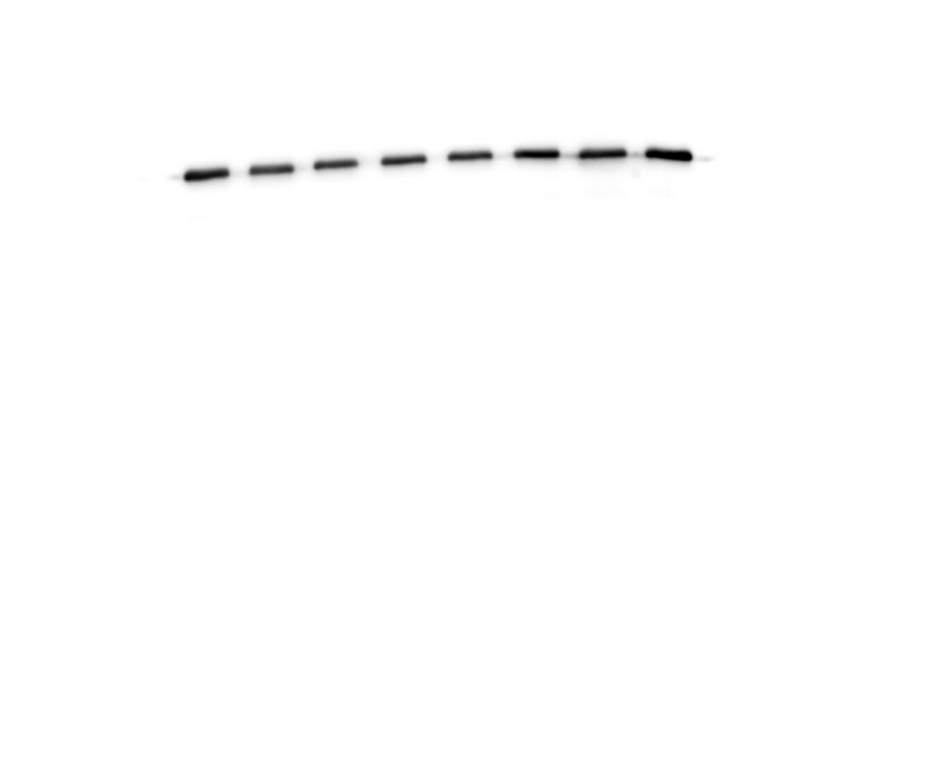 | 36 |
| 10 | p-PI3K | 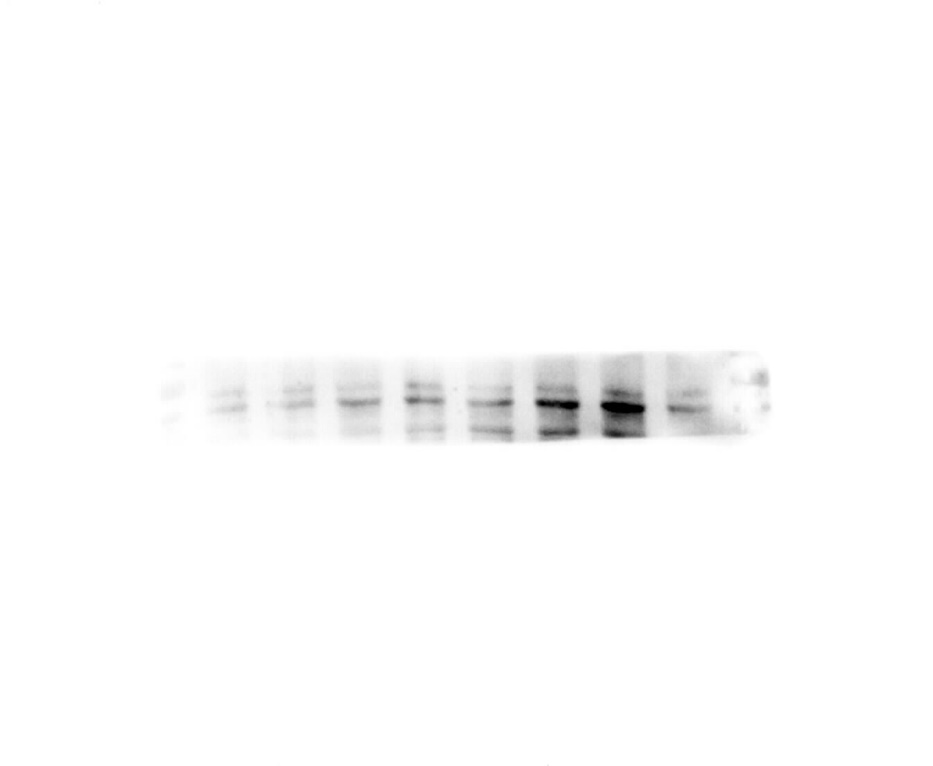 | 85 |
|  | PI3K | 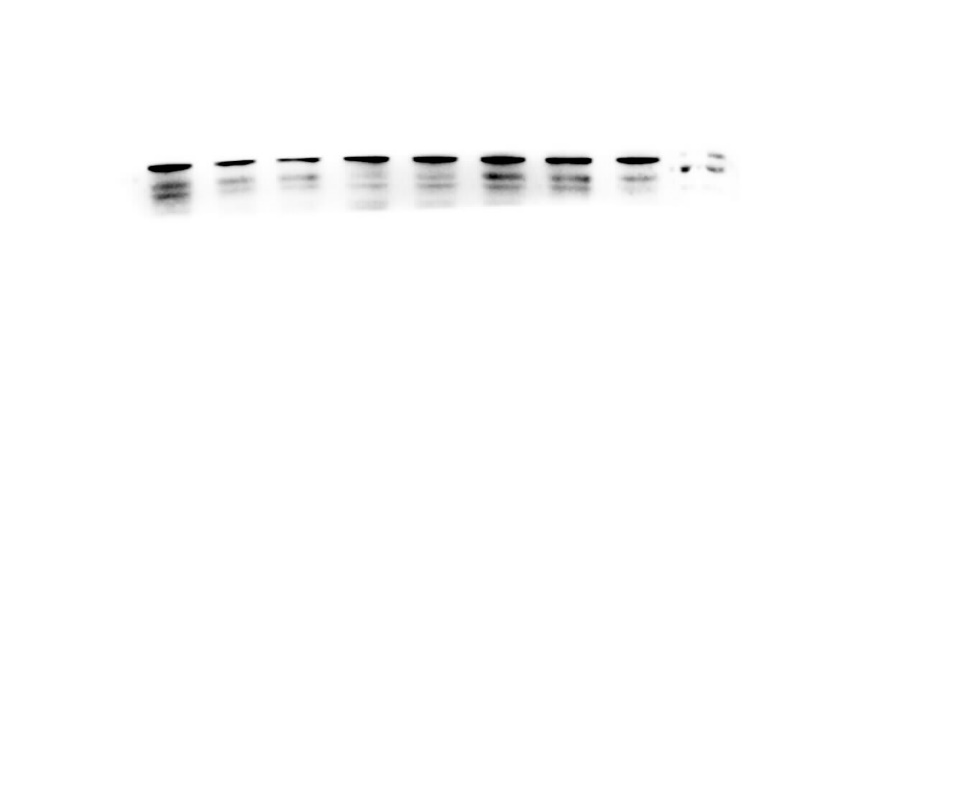 | 85 |
|  | GAPDH | 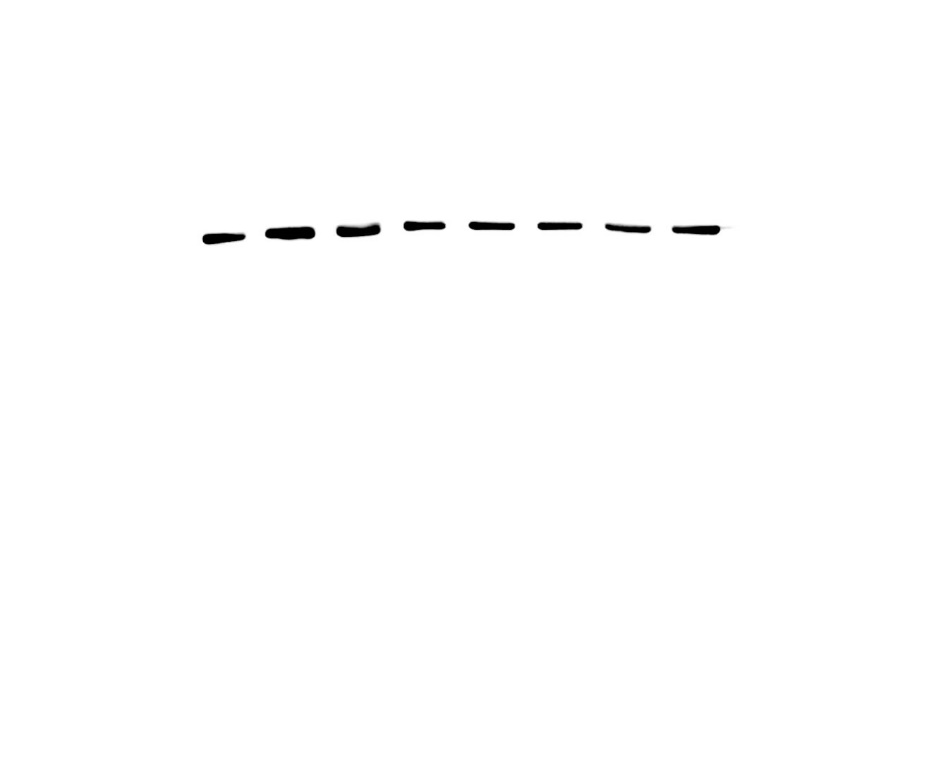 | 36 |
| 11 | p-AKT | 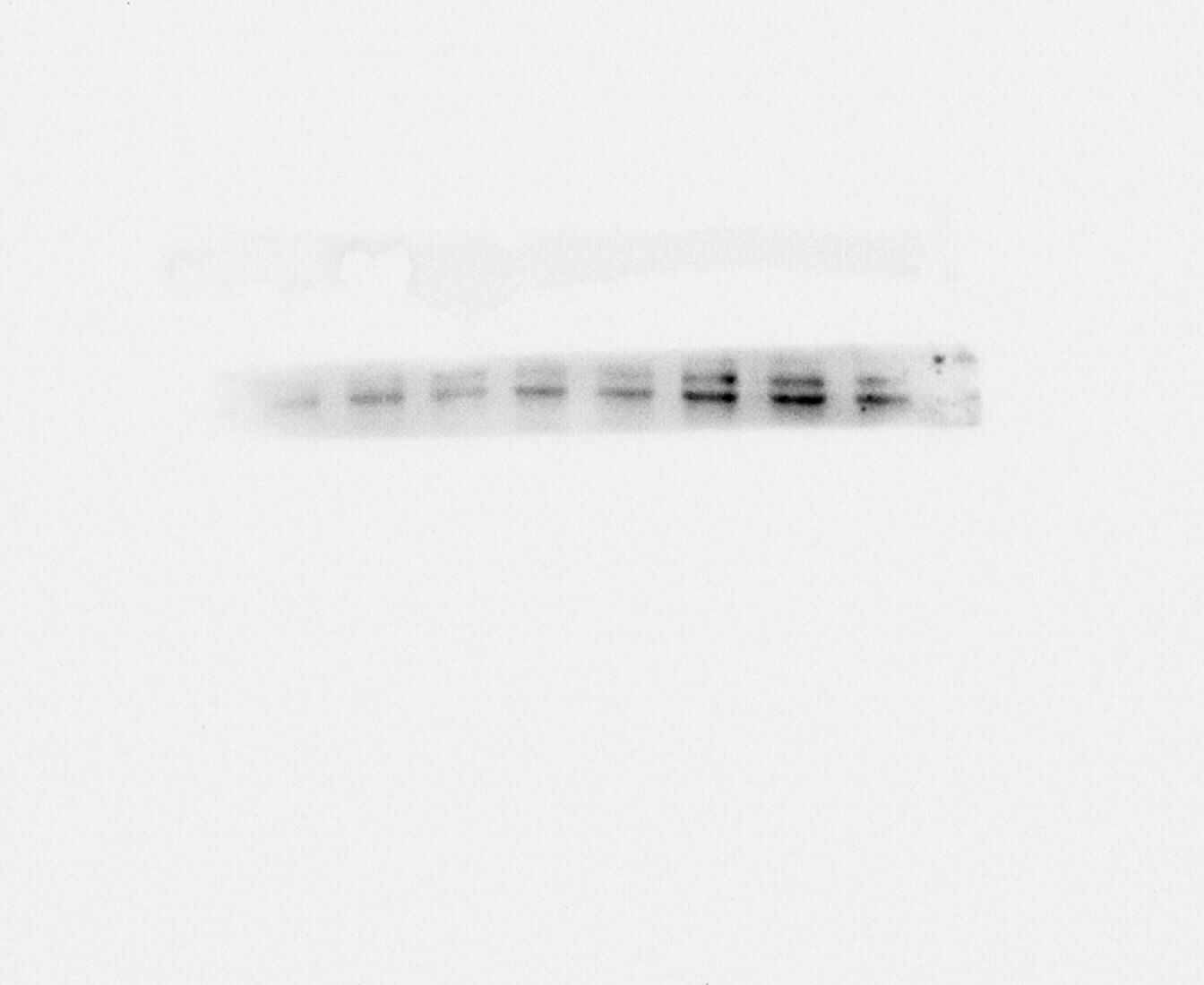 | 60 |
|  | AKT | 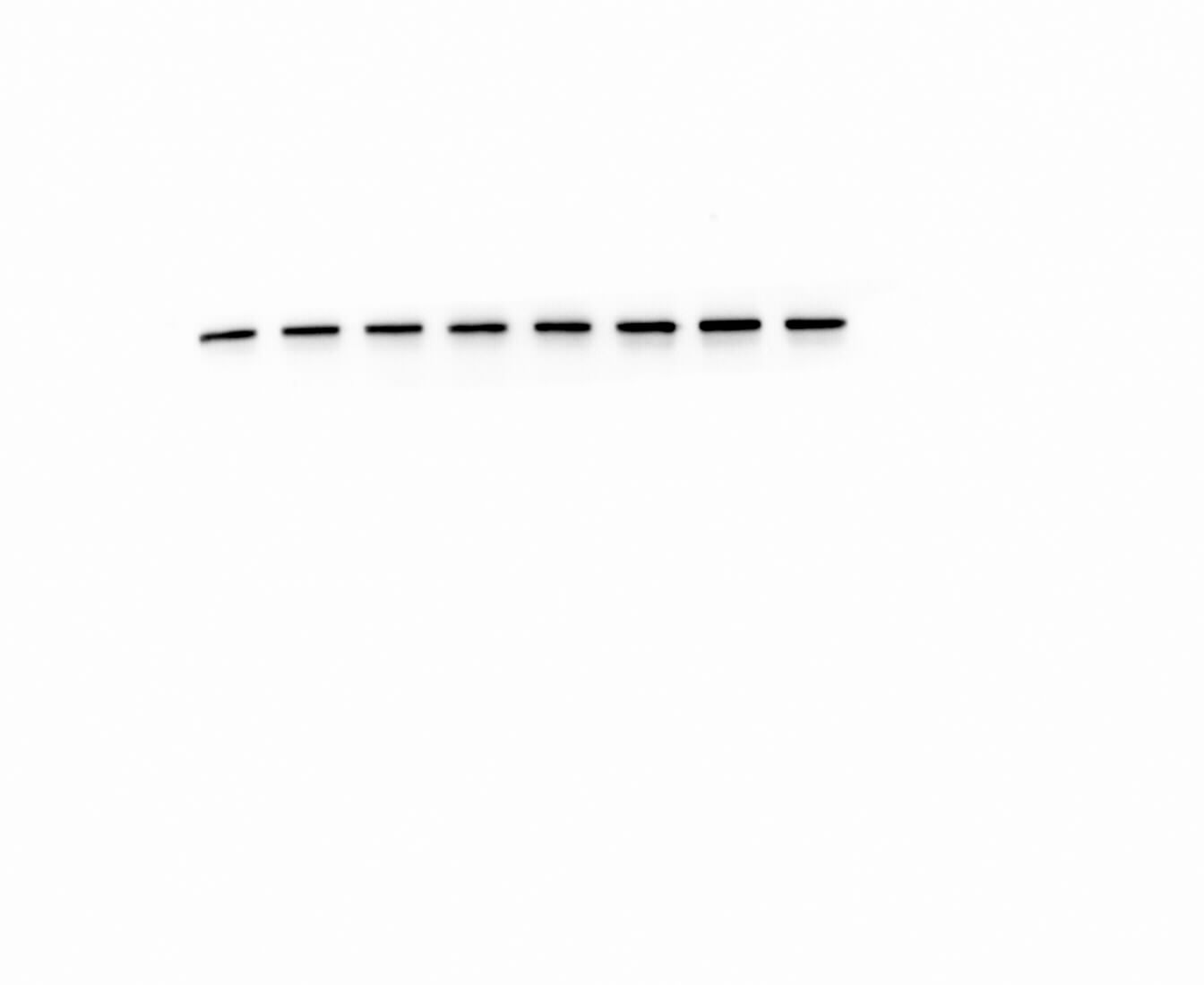 | 60 |
|  | GAPDH | 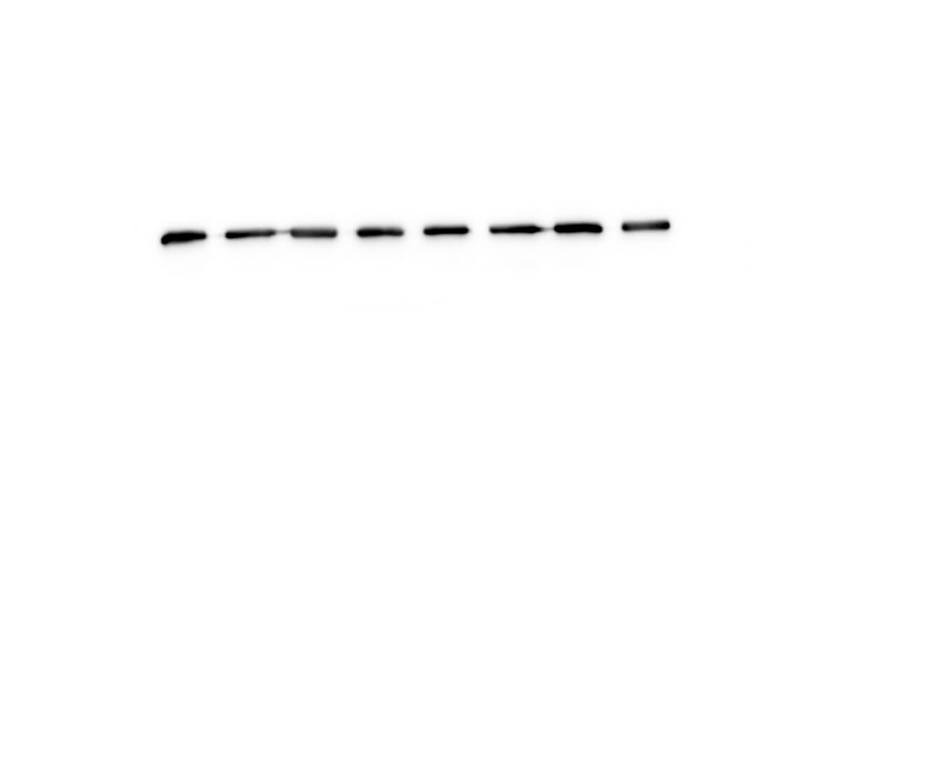 | 36 |
